# Human *MAMLD1* Gene Variations Seem Not Sufficient to Explain a 46,XY DSD Phenotype

**DOI:** 10.1371/journal.pone.0142831

**Published:** 2015-11-16

**Authors:** Núria Camats, Mónica Fernández-Cancio, Laura Audí, Primus E. Mullis, Francisca Moreno, Isabel González Casado, Juan Pedro López-Siguero, Raquel Corripio, José Antonio Bermúdez de la Vega, José Antonio Blanco, Christa E. Flück

**Affiliations:** 1 Pediatric Endocrinology and Diabetology, Department of Clinical Research, University Children’s Hospital Bern, Bern, Switzerland; 2 Pediatric Endocrinology Research Unit. Vall d’Hebron Institut de Recerca, Hospital Universitari Vall d'Hebron, Universitat Autònoma de Barcelona, CIBERER (Center for Biomedical Research on Rare Diseases), Instituto de Salud Carlos III, Barcelona, Spain; 3 Pediatric Endocrinology, Hospital Infantil La Fe, Valencia, Spain; 4 Pediatric Endocrinology, Hospital Universitario La Paz, Madrid, Spain; 5 Pediatric Endocrinology, Hospital Materno-Infantil, Málaga, Spain; 6 Pediatric Endocrinology, Corporació Parc Taulí, Hospital de Sabadell, Sabadell, Spain; 7 Pediatric Endocrinology, Hospital Universitario Virgen Macarena, Sevilla, Spain; 8 Pediatric Urology, Hospital Universitari Germans Trias i Pujol, Badalona, Spain; Virginia Commonwealth University, UNITED STATES

## Abstract

*MAMLD1* is thought to cause disordered sex development in 46,XY patients. But its role is controversial because some *MAMLD1* variants are also detected in normal individuals, several *MAMLD1* mutations have wild-type activity in functional tests, and the male *Mamld1*-knockout mouse has normal genitalia and reproduction. Our aim was to search for *MAMLD1* variations in 108 46,XY patients with disordered sex development, and to test them functionally. We detected *MAMDL1* variations and compared SNP frequencies in controls and patients. We tested MAMLD1 transcriptional activity on promoters involved in sex development and assessed the effect of MAMLD1 on androgen production. MAMLD1 expression in normal steroid-producing tissues and mutant MAMLD1 protein expression were also assessed. Nine *MAMLD1* mutations (7 novel) were characterized. *In vitro*, most MAMLD1 variants acted similarly to wild type. Only the L210X mutation showed loss of function in all tests. We detected no effect of wild-type or MAMLD1 variants on CYP17A1 enzyme activity in our cell experiments, and Western blots revealed no significant differences for MAMLD1 protein expression. MAMLD1 was expressed in human adult testes and adrenals. In conclusion, our data support the notion that *MAMLD1* sequence variations may not suffice to explain the phenotype in carriers and that MAMLD1 may also have a role in adult life.

## Introduction

The *MAMLD1* (mastermind-like domain-containing 1, previously also known as *CXorf6*) gene (OMIM 300120) has been first identified in a patient with myotubular myopathy and male hypogenitalism who was found to harbor a deletion on chromosome Xq28 [1, 2]. Meanwhile, 20 *MAMLD1* sequence variations have been described in patients who have a 46,XY disorder of sex development (DSD), mostly presenting with hypospadias [3–9]. However, other forms of DSD are also reported with *MAMLD1* gene mutations. Some patients present with cryptorchidism [3, 4, 6] and/or microphallus [6, 7]. Recently, the *MAMLD1* gene variation P677L was found in a 46,XY patient with complete gonadal dysgenesis [10]. In addition, two 46,XY DSD brothers with the *MAMLD1* mutation Q580R presented with female external genitalia. But strikingly, their nephew with the same severe 46,XY DSD phenotype did not carry the mutation [3]. By contrast, only one homozygous *MAMLD1* mutation (V505A) has been reported in a 46,XX DSD subject with gonadal dysgenesis [11]. She presented with primary amenorrhea, eunuchoidism, clitoromegaly and bilateral streak gonads [11].

Presently, there is some controversy about the causative role of *MAMLD1* gene variations and the associated DSD phenotype in carriers for the following reasons. First, some *MAMLD1* variants have also been detected in normal individuals (P359S, V505A, N662S) [3, 5, 7, 12] and others are not present in all affected DSD individuals of the same family (P359S, Q580R) [3]. Second, the male *Mamld1* knockout mouse has a normal genital phenotype and has normal reproduction [13]. Third, functional studies *in vitro* show normal results for several *MAMLD1* mutations compared to wild type (WT) when studying their effect as suggested regulators of genes involved in sex development [5, 14]. Finally, *MAMLD1* variants are also found in other species such as dogs, cats and horses with or without DSD [15–17].

Moreover, the V505A MAMLD1 variant has been found in the WT genome of the homo Neanderthal and chimpanzee [18]. This variant is regarded as an ancestral, potentially compensated mutation which is only disease-causing in humans [18]. Nevertheless, analyses of large case-control studies revealed the double S-S haplotype including MAMLD1 P359S and N662S as a risk factor for hypospadias [5, 7].

Little is known about the exact role of MAMLD1 in sexual development. A role in sex differentiation through supporting testosterone production in critical periods of male development has been suggested [14]. Studies in mice revealed increasing *Mamld1* expression from E12.5 to E14.5 in fetal Leydig and Sertoli cells [13]. *MAMLD1* is controlled by SF-1 which is a key transcription factor for numerous genes involved in sex development and steroidogenesis [14]. MAMLD1 transactivates also the non-canonical Notch targeted *Hes3* promoter [14]. *Hes3* regulates cell differentiation and proliferation during embryonic development [19]. MAMLD1 seems related to the production of testosterone as its knock-down reduces testosterone production and gene expression of *CYP17A1* [20]. Studies in *Mamld1*-KO mice showed significantly reduced testicular expression of Leydig-specific genes such as *Star*, *Cyp11a1*, *Cyp17a1*, *Hsd3b1* and *Insl3*, but normal expression of other genes related to steroidogenesis and sex-development [13]. However, *Mamld1*-KO mice have normal external genitalia and are able to reproduce similar to WT animals [13]. Taken together, there is justified doubt whether *MAMLD1* gene variations are sufficient to explain the DSD phenotype in carriers warranting further studies.

In this study we searched for *MAMLD1* sequence variations in a cohort of 108 46,XY DSD individuals in whom mutations in other candidate genes (*AR*, *SRD5A2*, *NR5A1*) were previously ruled out. We found 9 *MAMLD1* mutations (7 of them novel) in 108 46,XY DSD patients (8.3%). Patients’ characteristics were compared to reported cases. *In vitro* functional studies revealed negative results for most *MAMLD1* variants. Comparative alignments showed that the original amino acids are mostly not conserved through evolution, yet V505 exists only in human. Overall, our data support the notion that *MAMLD1* sequence variations may not suffice to explain the DSD phenotype in carriers.

## Methods

### Patients and genetic analyses

A cohort of 108 46,XY DSD individuals in whom mutations in other candidate genes (*AR*, *SRD5A2*, *NR5A1*) were previously ruled out was analyzed for *MAMLD1* sequence variation. Written informed consent was obtained from all individuals participants/legal guardians included in the study after full explanation of the purpose and nature of all the procedures used. Each patient’s pediatric endocrinologist provided the clinical and biochemical data. The genetic analyses were performed at the Vall d’Hebron Research Institute in Barcelona and the *in vitro* and *in silico* functional studies were done at the Pediatric Endocrinology Research laboratory in Bern. The molecular studies were approved by the ethic committees of the Vall d’Hebron Research Institute, Barcelona, Spain and the Ethic Commission of the Kanton Bern, Switzerland. Data entering the study were provided by the clinicians and the genetic lab in coded forms and are stored and accessible to the scientific community as follows: a) requests for clinical and biochemical data may be addressed to specific clinicians by contacting the corresponding author, b) genetic data are accessible through the Biobank system of Vall d’Hebron (biobanc@vhir.org), c) relevant experimental data are provided as a Supporting file (S1 File). The methods were carried out in accordance with the approved guidelines. Genomic DNA was isolated from peripheral blood leukocytes. *MAMLD1* coding regions, their flanking intronic sequences and part of the 5’UTR were amplified by PCR using specific primers [S1 Table, [4]]. The PCR products were sequenced using the BigDye Terminator v3.1 Cycle Sequencing Kit on an automated ABI PRISM 3100 Genetic Analyzer (Applied Biosystems, Foster City, CA, USA). Obtained sequences were analyzed against GenBank entries NG_017093.2 (genomic DNA), NM_005491.4 (mRNA) and NP_005482.2 (protein) (http://www.ncbi.nlm.nih.gov/). Genetic analyses of the *AR*, *SRD5A2* and *NR5A1* genes were performed as described [21–23]. All patients were checked for these 3 genes except patient 5, which was analyzed for *NR5A1* only.

A *MAMLD1* SNP genotyping (rs62641609: H347Q; rs41313406: P359S; rs61740566: V505A and rs2073043: N662S) of 155 normal adult male controls was performed by TaqMan assays for allelic discrimination using the Applied Biosystem Prism 7900 HT instrument and the allelic discrimination end-point analysis mode of the Sequence Detection software package, Version 2.3 (SDS 2.3). The following custom TaqMan SNP genotyping assays were used according to the protocols supplied by Applied Biosystems: C__64647092_10; C__26000187_10; C__25995288_20; C__15950293_10. Differences between controls and patients for SNP genotype and allele frequencies were analyzed with a contingency table analysis using the JMP^®^7 program (SAS Institute, Inc., Cary, NC, USA).

### Tissue expression studies

MAMLD1 expression was studied for normal human adrenal and testicular tissues using cDNA samples purchased from amsbio (AMS Biotechnology (Europe) Limited, Abingdon, UK). According to amsbio information, cDNAs originate from two adult subjects aged 50 years for adrenals and aged 23 years for testes. Fetal material was from two samples 20 weeks gestation for adrenals and 30 weeks gestation for testes. Semiquantitative PCRs (35 cycles) for MAMLD1 expression were carried out using the recommended concentrations of the purchased cDNAs. Primers are listed in S1 Table. Agarose-gel electrophoresis was performed for the PCR products, which were detected by ethidium bromide on a UV transilluminator (Alphaimager, Proteinsimple, Santa Clara, CA, USA).

### *In vitro* functional and expression studies

#### Cell lines

Steroidogenic cell lines mouse Leydig MA-10 (ATCC CRL-305; http://www.lgcstandards-atcc.org), human adrenal NCI-H295R (ATCC CRL-2128; http://www.lgcstandards-atcc.org) and the non-steroidogenic, human embryonic kidney HEK293 cell line (ATCC CRL-1573; http://www.lgcstandards-atcc.org) were used for this study. HEK293 cells were cultured in DMEM, supplemented with 10% fetal calf serum, 1% penicillin/streptomycin (Gibco, Paisley, UK) and 1% sodium pyruvate (Gibco). NCI-H295R cells were cultured in DMEM/Ham’s F12 (1:1) (Gibco), supplemented with 5% Nu serum (Becton Dickinson AG, Allschwil, Switzerland), 1% penicillin/streptomycin and 0.1% ITS Premix (Becton Dickinson). MA-10 cells were cultured in Waymouth MB 751/1 (Sigma-Aldrich Corp., St. Louis, MO, USA) supplemented with 15% Horse Serum (Gibco) and 1% penicillin/streptomycin (Gibco).

#### Expression vectors

The promoter luciferase reporter vector Hes3_luc (-2715~+261) was kindly gifted by Dr. Maki Fukami (National Research Institute for Child Health and Development, Tokyo, Japan) [14, 24]. The human -3.7CYP17A1_Δluc vector was available from previous work [25, 26]. For the MAMLD1 expression vectors, we used the WT cMyc-MAMDL1_pCMV and the minor transcript variant lacking exon 5 ΔE5 (previous ΔE4), both kindly gifted by Dr. Maki Fukami. In addition, we modified the WT cMyc-MAMLD1_pCMV vector according to the revised coding sequence NM_005491.4 (GenBank MAMLD1, isoform 2, new transcription start site in r.64, exon 2). This new WT construct was custom made by GenScript (Piscataway, NJ, USA). Mutant MAMLD1 expression vectors (c.605C>T, c.626delT, c.631G>A, c.1041C>A, c.1075C>T, c.1508C>A, c.1514T>C, c.1985A>G, c.2170C>G and c.2190G>A) were generated by PCR-based site-directed mutagenesis using specific primers (S1 Table) and the QuickChange protocol by Stratagene (Agilent Technologies Inc., Santa Clara, CA, USA) using the new WT expression vector as template. The MAMLD1 mutant c.1503_1504dupCAGCAG was also custom made by GenScript. All new constructs were verified by direct sequencing.

#### MAMLD1 promoter transactivation experiments

Cells were cultured on 24-well plates and transiently transfected (Lipofectamine 2000TM, Invitrogen) with WT or mutant MAMLD1 together with the promoter luciferase reporter constructs Hes3_luc or -3.7CYP17A1_Δluc for 6 hours. The transfection mixture contained 1.25 μg of plasmid DNA and 50 ng *Renilla* of luciferase reporter (pRL-TK) control per well. Forty-eight hours after transfection, cells were washed with PBS, lysed and assayed for luciferase activity with the Dual-Luciferase Reporter (DLR^™^) Assay System (Promega AG, Wallisellen, Switzerland) on a Veritasmicroplate Luminometer reader (Turner BioSystems Luminometer and Software by Promega). *Firefly* luciferase readings were standardized against *Renilla* control readings and results expressed as relative luciferase units (RLU). Experiments were performed in duplicates and repeated 3 times. Data are given as mean±SEM. Statistical significance was examined using Student’s t-test (Microsoft Excel). P-value for significance was set at ≤0.05.

#### CYP17A1 enzyme conversion experiments

To measure a possible effect of MAMLD1 on CYP17A1 activity, we assessed the conversion of progesterone to 17-hydroxyprogesterone and androstenedione in steroidogenic NCI-H295R and MA-10, and non-steroidogenic HEK293 cells. For this, all cells were transfected with WT or mutant MAMLD1 and non-steroidogenic HEK293 cells in addition with human CYP17A1 in pcDNA3. Experiments were performed on 12-well plates; transient transfection was for 6 hours (1.25 μg plasmid DNA/well) and experiments were closed after 48 hours. Steroid conversion was labeled by adding 20,000 cpm^14^C-progesterone per well for 60 min before extracting all steroids from the cell medium. Steroids were then separated by thin layer chromatography (TLC; Macherey-Nagel, Düren, Germany) using the chloroform:ethylacetate (3:1) solvent system and steroid standards. TLC plates were exposed on imaging screens to visualize the radioactive steroids. The screens were read on a Fuji PhosphoImager Fla-7000 (Fujifilm, Dielsdorf, Germany). Steroids were identified according to known stardards and densitometrically quantified as % of total radioactivity per sample using Multi Gauge software (Fujifilm). Experiments were performed 2 times in 3 different cell line backgrounds.

#### MAMLD1 protein expression studies

Leydig MA-10 cells were transiently transfected with either WT (WT, original WT of ΔE5) or mutant MAMLD1 expression vectors, which all carried a Myc-tag. Cells were lysed and a Western blot was performed using an antibody against c-Myc (C6594, Sigma, Saint Louis, USA). Experiments were repeated twice. Expression of β-actin was the control.

### *In silico* analysis

The following databases and computational tools were used. CLC Sequence Viewer software (2014 CLC bio, QIAGEN) was used to search for homologies of MAMLD1 through species. Polymorphism Phenotyping v2 (PolyPhen-2, http://genetics.bwh.harvard.edu/pph2/index.shtml) [27] was used to predict the possible impact of amino acid substitutions on the structure and function of MAMLD1. We searched for functional partners of MAMLD1 with the Search Tool for the Retrieval of Interacting Genes/Proteins (STRING, http://string-db.org/), developed at the Center for Protein Research (CPR), EMBL, Swiss Institute of Bioinformatics (SIB), University of Copenhagen (KU), Technical University of Dresden (TUD) and University of Zurich (UZH). The Biological General Repository for Interaction Datasets (BioGRID, thebiogrid.org), developed at Princeton University, University of Montreal, University of Edinburgh and Mount Sinai Hospital, is a public database that was searched for protein interactions.

## Results

### Patients’ genotypes and phenotypes

**Fig 1:**
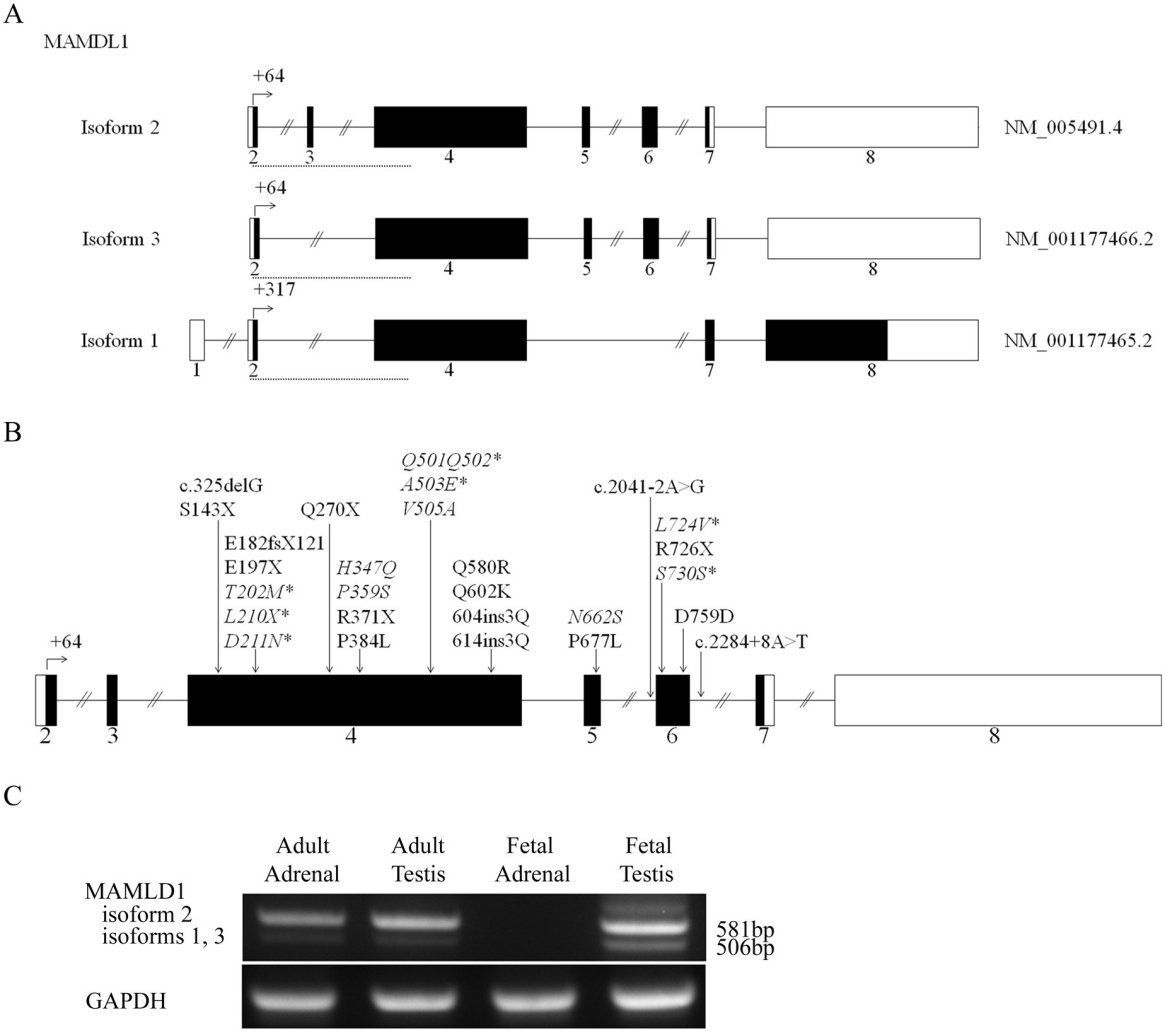
MAMLD1 transcripts, reported mutations and tissue expression. A. Schemes of the 3 MAMLD1 human transcripts are shown (http://www.ncbi.nlm.nih.gov/; http://www.ensembl.org). B. Scheme showing all reported *MAMLD1* gene mutations. Mutations described in this study are shown in red and the novel ones are marked with an asterisk. C. Assessment of MAMLD1 expression in human fetal and adult adrenal and testis. Semiquantitative RT-PCRs were performed using specific primers (S1 Table). GAPDH was used as the internal control. A representative gel picture is shown (n = 3). For MAMLD1 the band at 581 bp corresponds to isoform 2 and the band at 506 bp to isoforms 1 and 3. Dashed red lines in A indicate the location of the PCR fragments amplified for the expression studies.

We searched for mutations in the MAMLD1 gene (Fig 1A) in a cohort of 108 46,XY DSD patients from Spain and Switzerland. *MAMLD1* is located on Xq28. The original *MAMLD1* gene sequence has been recently revised [6] for a new upstream transcriptional start site (TSS): +64 (exon 2) (old TSS +284) which has caused a renumbering of exons and sequence (http://www.ncbi.nlm.nih.gov/gene/10046) and a renaming of the previously identified mutations (Fig 1). MAMLD1 presents with 3 different isoforms (Fig 1A). Isoform 2 (NM_005491.4, NP_005482, ENST00000262858, 774 amino acids), which is coded by exons 2 to 7, is considered the canonical sequence and has been studied previously [3, 6, 10, 11, 14]. Isoform 3 (NM_001177466, NP_001170937, ENST00000426613, 749 amino acids) is identical to isoform 2, but lacks exon 3. Isoform 1 (NM_001177465, NP_001170936, ENST00000432680, 998 amino acids) has a non-coding upstream codon, lacks exon 3, and has a different C-terminus compared to isoforms 2 and 3 due to a translational frameshift after exon 4.

In our cohort of 46,XY DSD a total of 9 sequence variations were detected (Fig 1B). Three *MAMLD1* sequence variations were considered SNPs, but had been previously described in both controls and 46,XY DSD individuals (rs41313406: c.1075C>T, P359S; rs61740566: c.1514T>C, V505A and rs2073043: c.1985A>G, N662S) [3–7, 14]; one was previously detected in 46,XY patients only (rs62641609: c.1041C>A, H347Q) [6], and 7 were novel (c.605C>T, p.T202M; c.626delT; p.L210X; c.631G>A, p.D211N; c.1503_1504dupCAGCAG, p.Q501Q502; c.1508C>A, p.A503E; c.2170C>G, p.L724V and c.2190G>A, p.S730S). Nine sequence variations found in 9 patients were considered potentially pathogenic. These patients manifested with a broad range of 46,XY DSD phenotype (Table 1). Six patients presented rather severe hypospadias, 2 also had cryptorchidism, and 1 presented with normal external female genitalia with gonads palpable in the genital folds. Four 46,XY subjects were reared as females, 5 as males. Testis histology was studied in 3 patients between age 2 weeks and 2 years and was normal. Hormonal studies for gonadal function demonstrated normal baseline and/or hCG stimulated testosterone production and normal AMH levels (Table 1). Subject 5 claimed to have fathered a child. Adrenal function tests were normal in all tested patients. Additional diagnoses were present in 3 patients, 1 related to a 22q11 deletion syndrome (subject 8, Table 1).

**Table 1:** Clinical, biochemical and genetic characteristics of the patients harboring mutations and polymorphisms in the *MAMLD1* gene.

Patient	Origin, YOB	Karyotype, Assigned sex	*MAMLD1* gene mutation	Genital anatomy	Testes histology (age)	Gonadal function (age)	Adrenal function (age)	Remarks
1	Spain, 2009	46,XY, Male	**T202M**, c.605C>T	Penoscrotal hypospadias. Small penis. Unilateral cryptorchidia.		Normal T (minipuberty). Normal gonadotropins. No hCG test.	ND	SGA. Short stature. Low implantation thumbs.
2	Spain, 1999	46,XY, Female	**L210X**, c.626delT and **D211N**, c.631G>A	Penoscrotal hypospadias. Small penis. Testes 0.5 ml.	Normal for age (15 d).	Normal T (baseline) and hCG test (10 days).	Normal baseline (15 d).	Abnormal GGN repeat in *AR*. Mother non-carrier; norfloxacine treatment during pregnancy.
3	Spain, 2005	46,XY, Female	**H347Q**, c.1041C>A, *rs62641609*	Female genitalia. Gonads in labia.		Normal hCG test (2 y).	Normal baseline (2 y).	
4	Spain, 2008	46,XY, Male	**H347Q**, c.1041C>A, *rs62641609*	Penoscrotal hypospadias. Testes 2 ml.		Normal hCG test. Normal AMH (2.5 y).	Normal baseline (2.5 y).	
5	Switzerland, 1942	46,XY, Male	**Q501Q502**, c.1503_1504dupCAGCAG	Hypospadias. Short penis. Delayed puberty. Testes 8 ml.		Baseline T and gonadotropins normal (70 y). Fathered a boy.	Normal baseline (70 y).	
6	Spain, 2001	46,XY, Male	**A503E**, c.1508C>A	Penoscrotal hypospadias. Small penis. Testes 2 ml.		Normal baseline T (3 m). Normal hCG test (9 m).	Normal baseline (3 d).	
7	Spain (Venezuelan origin), 2000	46,XY, Female	**V505A**, c.1514T>C, *rs61740566*	Penoscrotal hypospadias. Small penis. Unilateral cryptorchidia.	Normal for age (2 y).	Normal hCG test.	Normal Synacthen test.	
8	Spain (North African origin), 2010	46,XY, Male	**L724V**, c.2170C>G	Penoscrotal hypospadias. Small penis. Testes 1 ml.		Normal prepubertal baseline T (15 m). Normal AMH.	Normal baseline (15 m).	Esophageal atresia. Right aortic arch.
9	Spain, 1990	46,XY, Female	**S730S**, c.2190G>A	Penoscrotal hypospadias. Small penis.	Normal for age (nests of Normal Leydig cells; normal fertility index (1 y).	Normal baseline (12 m). No hCG test.		Müllerian ducts.

ND: not done. d: day(s), m: month(s), y: year(s).

### *MAMLD1* polymorphism testing

We chose 4 *MAMLD1* variants (H347Q, P359S, V505A and N662S), previously detected in controls and 46,XY DSD patients [3, 5–7] and/or referenced in dbSNP (http://www.ncbi.nlm.nih.gov/snp/) to compare their genotype and allele frequency in a cohort of 155 normal adult male controls (Table 2) and our cohort of 108 46,XY patients (Table 2) (with phenotypes varying from penoscrotal hypospadias to female external genitalia and including the 9 in whom *MAMLD1* sequence variations were detected, Table 1). The MAMLD1 variants H347Q and V505A were not found in our 155 normal adult male controls suggesting a causative role for these genetic variations; the H347Q variant was not present in two exome pools of the European population (http://evs.gs.washington.edu/EVS/ and.http://exac.broadinstitute.org/about), but the V505A was detected in 0.05% and 0.1% of these European subjects, and in 17.8% of African people (http://exac.broadinstitute.org/about). The variants P359S and N662S were also quite frequently found in our controls (Table 2), and frequencies were similar in the databases. Linkage analysis of variants P359S and N662S was positive for 84% and 92% of controls and 46,XY DSD carrying the N662S variant (Table 2), thus indicating high co-segregation of both variants.

**Table 2:** Study of polymorphisms in the *MAMLD1* gene.

		Controls (n = 155)	46,XY DSD (n = 108)
Sequence change, **NM_005491.3** (NT_167198.1/ U46023)	Allele	Frequency (n)	Frequency (n)
**H347Q** (H274Q) *rs62641609*	C	1.00 (155)	0.98 (106)
	A	0.00 (0)	0.02 (2)
**P359S** (P286S) *rs41313406*	C	0.85 (134)	0.90 (97)
	T	0.15 (21)	0.10 (11)
**V505A** (V423A) *rs61740566*	T	1.00 (155)	0.99 (107)
	C	0.00 (0)	0.01 (1)
**N662S** (N589S) *rs2073043*	A	0.84 (130)	0.89 (96)
	G	0.16 (25)	0.11 (12)

### MAMLD1 tissue expression

We studied MAMLD1 expression for human fetal and adult adrenal and testis tissues. We found that MAMLD1 is expressed in the adult adrenal and testis, and the fetal testis. No expression was found in the fetal adrenal (Fig 1C). We detected all 3 isoforms in similar quantities.

### *In vitro* and functional studies

**Fig 2:**
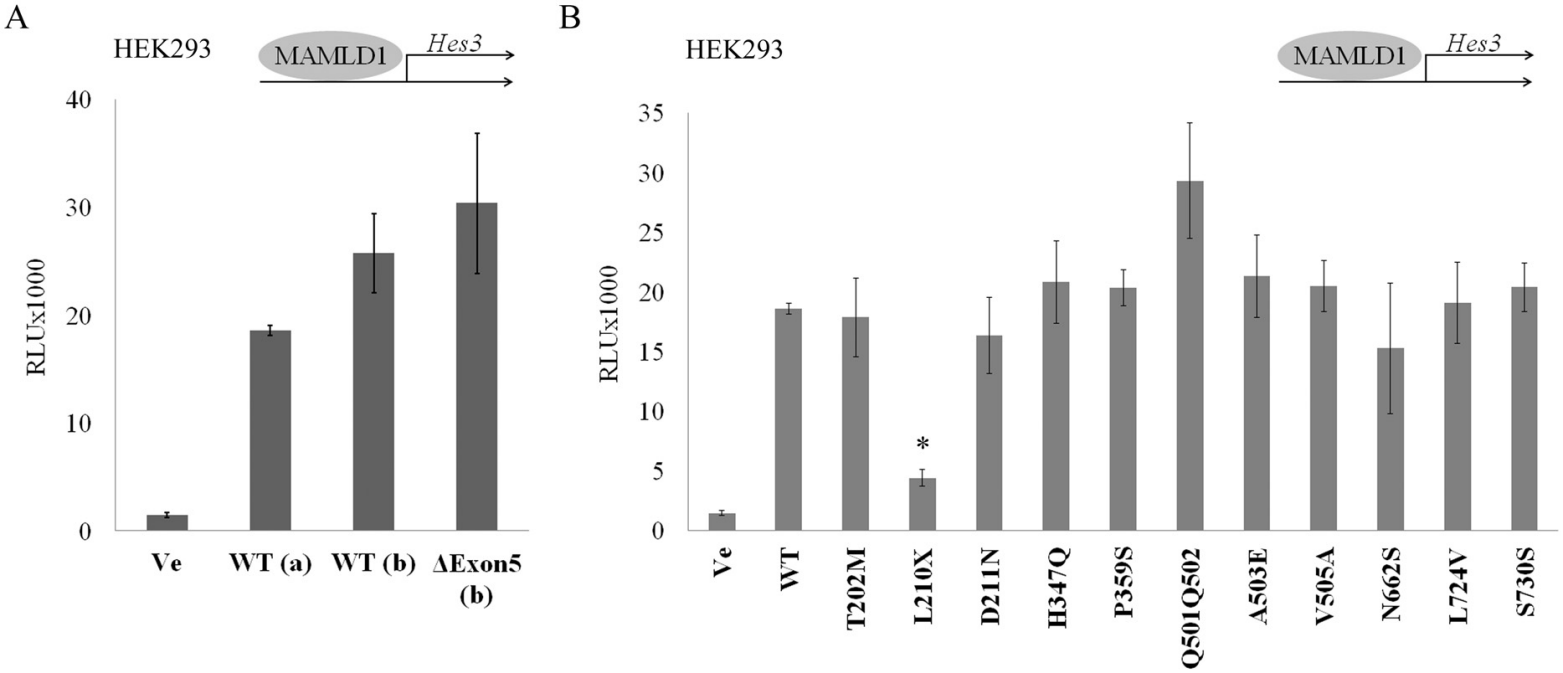
Transactivation activity of MAMLD1 on the *Hes3* promoter. HEK293 cells were transiently transfected with wild-type (WT) and mutant MAMLD1 expression vectors and with a *Hes3* promoter luciferase reporter construct. Luciferase activity was measured with the Promega Dual Luciferase assay system. A. Comparison of the newly constructed MAMLD1 WT expression vector (WT (a), NM_005491.4) with the older WT (WT (b)), and ΔE5 (ΔE5 (b)) constructs [14]. Similar transactivation activity on the *Hes3* promoter was found for all constructs. B. *Hes3* transactivation by WT and the 11 MAMLD1 mutants was assessed. Only the L210X MAMLD1 mutant showed an impaired activity on the *Hes3* promoter. Results are expressed in relative light units (RLU) and represent the mean and SEM of 3 independent experiments performed in duplicate. ΔE5: original WT (b) without exon 5 [14]; * *p*≤0.05.

In 2012 the reference sequence of *MAMLD1* has been revised (GenBank NM_005491.4, [6]). Therefore, we modified the human MAMLD1 expression plasmid which has been originally used for functional studies in all published studies accordingly. We then first performed promoter transactivation studies (Fig 2) with the *Hes3* promoter reporter in non-steroidogenic HEK293 cells to compare the activity of the revised MAMLD1 WT vector with the original WT (Fig 2A). We found similar transactivation activity on the *Hes3* promoter construct for both WT isoforms (revised and original) as well as for the revised shorter isoform without exon 5.

We then created mutant MAMLD1 expression vectors by site-directed mutagenesis according to identified sequence variations in our 46,XY DSD patients. For unclear test results in the literature [3, 5, 7, 14], we added MAMLD1 P359S and N662S to our test series. The ability of these 11 MAMLD1 variants to transactivate the *Hes3* promoter in HEK293 cells was then assessed (Fig 2B). Surprisingly, only the L210X mutant lost transcriptional activity compared to WT.

**Fig 3:**
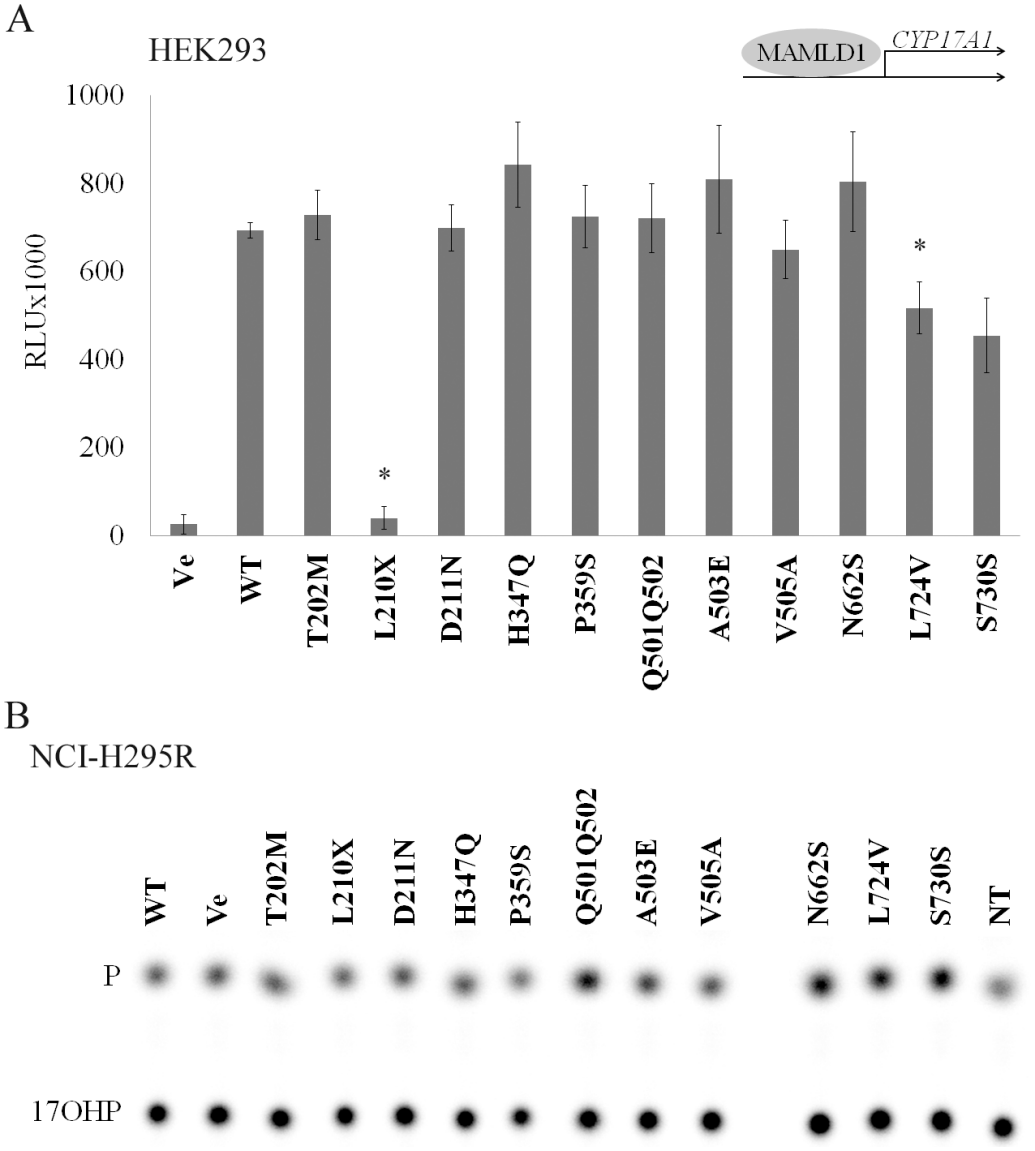
Effect of MAMLD1 on CYP17A1 promoter and enzyme activities. HEK293 cells or NCI-H295R cells were transiently transfected with MAMLD1 WT and mutant expression vectors. For promoter activation studies, the (-3.7kb) *CYP17A1* promoter luciferase reporter construct was co-transfected. A. *CYP17A1* promoter activation by MAMLD1 was assessed by the Promega Dual luciferase assay in HEK293 cells. Only for mutant MAMLD1 L210X and L724V an impaired *CYP17A1* activation was found. Results are expressed in RLU and represent the mean and SEM of 3 independent experiments performed in duplicate. B. The effect of WT and mutant MAMLD1 on CYP17A1 enzyme activity was assessed in transfected NCI-H295R, MA-10 and HEK293 cells by measuring the conversion of progesterone to 17-hydroxyprogesterone. Steroid production was labeled with [^14^C]progesterone for 60 min. Steroids were extracted and resolved by thin-layer chromatography, then quantified as % conversion. A representative steroid profile obtained from NCI-H295R cells is shown (n = 2). No effect of MAMLD1 on CYP17A1-hydroxylase activity was detected. P: progesterone; 17OHP: 17-hydroxyprogesterone; RLU: relative light units; Ve: empty vector; WT: wild type; NT: non-transfected; * *p*≤0.05.

Given these results, we performed similar studies with the human *CYP17A1* promoter (Fig 3), as studies in mice revealed an effect of MAMLD1 on CYP17A1 expression, activity and testosterone production [13, 20]. Again, most MAMLD1 variants showed WT effect on the *CYP17A1* promoter reporter (Fig 3A). Similar to the *Hes3* promoter, mutant L210X showed a loss of activity on the *CYP17A1* promoter, while variants L724V and S730S showed a decrease in promoter activation compared to WT (*p* = 0.052 for S730S).

Next, we also tested the effect of WT and MAMLD1 variants on CYP17A1 enzymatic activity. For that we transfected MAMLD1 expression vectors into human adrenal NCI-H295R and into mouse Leydig MA-10 cells. We also co-transfected MAMLD1 expression vectors together with a CYP17A1 expression vector into non-steroidogenic HEK293 cells. After transfection, activity of CYP17A1 was assessed by measuring the conversion of radiolabeled progesterone to 17-hydroxyprogesterone (Fig 3B, S2 Fig). We found no difference for CYP17A1 activity for WT MAMLD1 and variants in all 3 cell systems (data for NCI-H295R cells in Fig 3B; data for MA-10 and HEK293 in S2 Fig). Remarkably, there was also no difference between the control vector and the WT MAMLD1 indicating that MAMLD1 does not regulate human CYP17A1 activity or that the effect is indirect through essential co-factors that were not present in the 3 cell systems used for our experiments.

**Fig 4:**
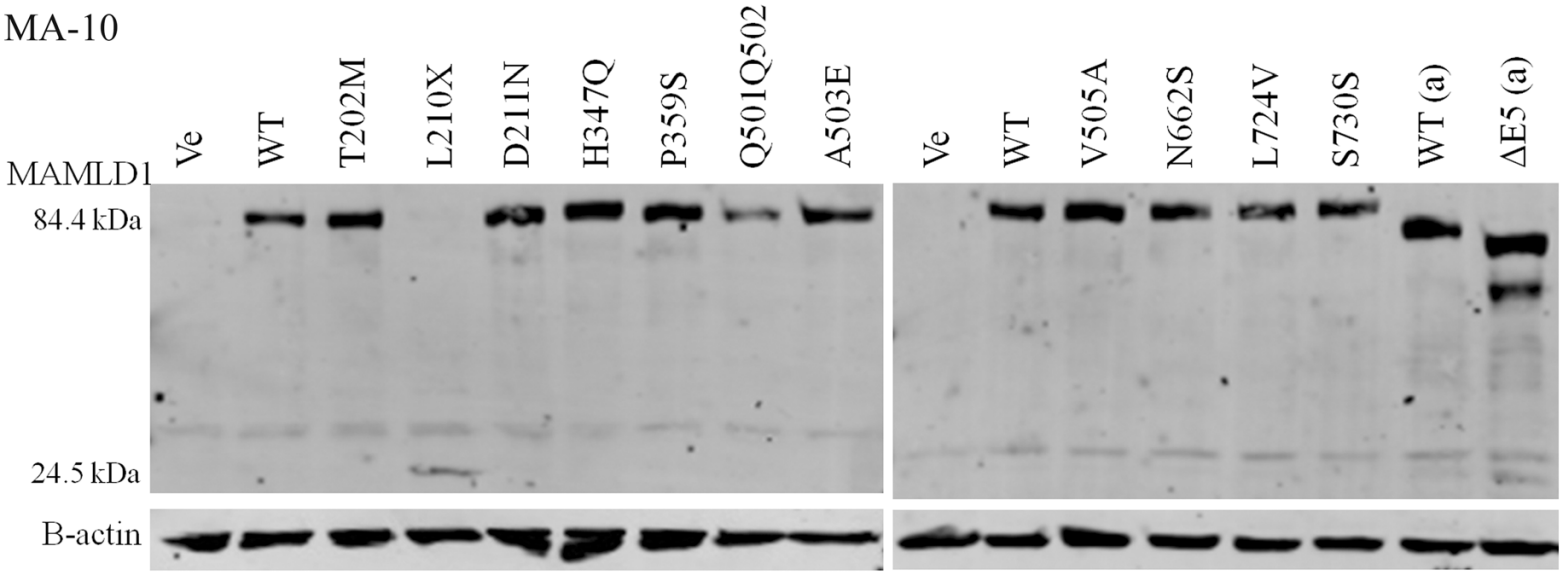
Protein expression of MAMLD1. Mouse testis Leydig MA-10 cells were transiently transfected with Myc-tagged expression vectors for WT or mutant MAMLD1. Cells were lysed and Western blot (WB) was performed using anti-Myc antibody. B-actin was the control. A representative WB is shown. Two independent experiments were performed showing no significant variation in protein expression for wild-type (WT) or mutant MAMLD1. L210X gave a shorter protein. Ve: empty vector; WT: wild-type; WT(a): original WT construct; ΔE5(a): original construct lacking exon 5 [14].

Some *MAMLD1* nonsense mutants have been suggested to affect protein expression by nonsense mediated RNA decay [E197X (old E124X), Q270X (old Q197X) and R726X (old R653X) [3]]. Therefore, we analyzed protein expression of WT and mutant MAMLD1 in MA-10 cells. Cells were transfected with the MAMLD1 expression vectors containing a Myc-tag, then Western blots were performed using an antibody against c-Myc (Fig 4). We found no significant difference for MAMLD1 protein expression for almost all of the tested variants compared to WT. The L210X mutant presented a lower band on the Western blot according to its shorter length and its amount may therefore not be compared to the full-length missense variants.

### *In silico* analyses

PolyPhen-2 (http://genetics.bwh.harvard.edu/pph2/index.shtml) [27] was used to predict the possible impact of amino acid substitutions due to missense mutations T202M, D211N, P359S, H347Q, A503E, V505A, N662S and L724V on the structure and function of MAMLD1 (NP_005482.2). Using standard parameters [27], only T202M, H347Q and L724V were considered probably damaging.

Variants T202M, D211N, H347Q, P359S and A503E are predicted to impact on protein function because the amino acid change comprises a change in the physico-chemical property. Proline is a bending amino acid, thus it may cause a change in the conformation of the protein and it is therefore considered important. According to this, P359 could have some effect on the protein function, although it is not a conserved position in mammals (S1 Fig).

Furthermore, we aligned the human MAMLD1 isoform 2 (774 amino acids) with homologous sequences (ranging 720–820 amino acids) from 40 mammalian species with the CLC Sequence Viewer software (S1 Fig). To study all detected species available in the NCBI database (e.g. beyond mammals) was not possible because of high variability in length. L210 and L724 (1 change each) are conserved along mammalian evolution, followed by S730 (deleted amino acid (-) in 2 species). They might therefore be of importance for protein function. The remaining variants’ positions T202, D211, P359, Q501Q502, A503, V505 and N662 are not conserved and therefore not considered important for protein function. Variants in positions 202, 347, 505 and 662 are WT in other mammals indicating that these amino acid changes may not be of importance. T202 and S202 are similarly found: T202 is present in one third of the species (primates and bovidae), while the other mammals present S202. For position 347, H is mainly present, but 2 other changes, including our variant Q347 (in 10 species), are seen. V505 is only present in human, whereas most other mammals harbor A505 (27 species), suggesting that human V505A may just be a reversion to the ancestral state. In position N662 4 variants are present including the variant S662. Finally, the duplication Q501Q502 lies in a Q sequence, which has variable length in different species. This indicates that the length change may not harm the protein function.

## Discussion

We detected 7 novel and 2 previously published *MAMLD1* sequence variations in 9 46,XY DSD patients presenting with a broad phenotype (Table 1). Similar to other studies, we found that for *MAMLD1* sequence variations genotype-phenotype correlation and functional studies reveal ambiguous results. In our series only the truncated *MAMLD1* L210X mutation, found in a severely affected 46,XY DSD patient, showed loss of function in transactivation assays using the *Hes3* and the *CYP17A1* promoters as interacting partner. L201X can therefore be considered a deleterious mutation. Remarkably, this patient also presented with hypogonadotropic hypogonadism at pubertal age suggesting that MAMLD1 may also be involved in the HPG axis, what has not been described so far, or that the patient may harbor additional genetic defects. By contrast, L724V, carried by a 46,XY DSD patient with a typical MAMLD1 phenotype (46,XY male with penoscrotal hypospadias and small penis), and the synonymous S730 mutation, found in 46,XY female patient (also with penoscrotal hypospadias and small penis), had only impaired *CYP17A1* but normal *Hes3* transactivation activity. Both positions are conserved in mammals. Although variant H347Q showed no functional impairment in our in vitro tests, its presence in 2 of our patients with a severe 46,XY DSD phenotype (female and ambiguous genitalia) and in a published 46,XY DSD patient with hypospadias and microphallus [6], as well as its absence in 155 control males suggests a disease causing role. Variant V505A, which we found in one patient, has been previously described in 2 46,XY DSD subjects [4, 7] and in a 46,XX dysgenetic woman [11]. Interestingly, this human variant is the WT found in the genome of the Neanderthal, the chimpanzee and many other species and has been discussed to be an ancestral, potentially compensated mutation [18].

According to published literature of microdeletions and rearrangements in the Xq28 region containing the MAMLD1-MTM1-MTMR1 genes [see Table 3 and [1, 2, 28–30]], which causes myopathy for the involvement of the MTM1 gene, it appears for the DSD phenotype that the C-terminal region of the MAMLD1 gene is critical for abnormal sex development [29]. By contrast, MAMLD1 variants which have been associated with DSD are found throughout the gene (Fig 1B).

**Table 3:** Summary of reported patients harboring sequence variations and deletions affecting *MAMLD1* gene ^a^.

Mutation number, Reference	Karyotype, Assigned sex	*MAMLD1* gene mutation	Genital anatomy	Gonadal function, Gonadal histology	Adrenal function	Family, Other data	Present in controls?	Functional/additional studies	Additional phenotype Other genetic studies
**1**. Metwalley and Farghaly, 2012 [8]	?, 1 male	c.325delG	Distal hypospadias with chordee and normal testes	Profile consistant with X-linked congenital adrenal hypoplasia	-	Egyptian origin	-	-	*Mutation in DAX-1* (R327P)
**2**. Kalfa *et al*., 2012 [6]	46,XY, 1 male	c.428C>A, S143X	Scrotal hypospadias, microphallus, intrascrotaltestis	Normal T, LH, FSH, AMH, Inhibin	-	Mother heteterozygous, uncle severe hypospadias, maternal cousin severe hypospadias (not available for testing)	-	Loss of transactivation activity on Hes3	N: *AR*, *SRD5A2*
**3**. Kalfa *et al*., 2008 [4]	?, 1 male	c.546del, E182fsX121	Proximal hypospadias, inguinal testis	NA	Normal		-	-	-
**3**. Kalfa *et al*., 2008 [4]	?, 1 male	c.546del, E182fsX121	Penoscrotal hypospadias with chordee, intrascrotal testis	NA	Normal		-	-	-
**4**. Fukami *et al*., 2006 [3]; Fukami *et al*., 2008 [14]	?, 2 males, half brothers	c.589G>T, E197X rs121909493	1^st^: penoscrotal hypospadias, inguinal testes; 2^nd^: penoscrotal hypospadias, scrotal testes; penis 2.5 cm	All normal, FSH low	Normal	Japanese origin. Mother heterozygous	Absent in 150 Japanese males	Causes non-mediated RNA decay. Reduced leukocyte transcripts. No transactivation activity on Hes3, reduced protein expression	N: *AR*, *SRD5A2*
**5**. Fukami *et al*., 2006 [3]; Fukami *et al*., 2008 [14]	?, 1 male	c.808C>T, Q270X rs121909494	Penoscrotal hypospadias, scrotal testes; penis 2 cm	Normal	Normal	Japanese origin. Mother not studied	Absent in 150 Japanese males	Causes non-mediated RNA decay. Reduced leukocyte transcripts. No transactivation activity on Hes3, increased protein expression	N: *AR*, *SRD5A2*
**6**. Kalfa *et al*., 2012 [6]	46,XY (1/70), 1 male	c.1041C>A, H347Q, rs62641609	Posterior hypospadias, microphallus	-	-	-	-	-	-
**7**. Kalfa *et al*., 2012 [6]	46,XY, 5 males	c.1075C>T, P359S, rs41313406	Non-syndromic DSD	-	-	-	-	-	-
**7**. Fukami *et al*., 2006 [3]; Fukami *et al*., 2008 [14]	?, 1 male	c.1075C>T, P359S, rs41313406	Hypospadias	NA	NA	Swedish origin. Absent in brother and nephew with same phenotype	In 8/110 Swedish Controls	Transactivation activity on Hes3 similar to WT	-
**7**. Chen *et al*., 2010 [7]	?, 11 males	c.1075C>T, P359S, rs41313406	Hypospadias	-	-	-	No	Weak association with hypospadias by screening in a case-control SNP-genotyping study	-
**7**. Kalfa *et al*., 2011 [5]	?, 17 males (17/150)	c.1075C>T, P359S, rs41313406	Hypospadias	-	-	-	Yes	Transactivation activity on Hes3 similar to WT	-
**8**. Lim *et al*., 2013 [31]	?, 1 male	c.1141C>T, R371X	Normal	-	-	-	Present in 1 control	Detected by exome sequencing in a study on autism	-
**9**. Kalfa *et al*., 2012 [6]	46,XY, 1 male	c.1151C>T, P384L	Penile hypospadias, microphallus, intrascrotal testis	Slightly low LH, FSH. Low T, AMH, inhibin	-	Maternal diabetes	-	Reduced transactivation activity on Hes3	N: *AR*, *SRD5A2*
**10**. Kalfa *et al*., 2008 [4]	?, 1 male	c.1514T>C, V505A, rs61740566	Isolated proximal hypospadias, intrascrotal testis	NA	NA	-	-	-	-
**10**. Zhang *et al*., 2010 [18]	?, Neandertal	c.1514T>C, V505A, rs61740566		-	-	-	-	-	-
**10**. Brandao *et al*., 2011 [11]	46,XX DSD (GD), 1 female	V505A, homozygote, rs61740566, (GOF)	Primary amenorrhea, no breast development, eunuchoid habitus, absence hirsutism, Tanner IV pubic hair, clitoromegaly, 2 perineal openings, unpalpable gonads. Bilateral streak gonads, small uterus, bilateral Fallopian tubes.	FSH eleated, normal LH, Prog, 17OH-Prog, androstenedione, T non increasing after hCG stimulation. Histology: absence of left gonad; fallopian tubes and a dysgenetic right gonad with hilar cell hyperplasia and persistence of Wolffian rests	-	Parents are first cousins	Absent in 190 normal alleles	Transactivation activity on Hes3 and Hes7 higher than WT	N: *FOXL2*, *BMP15*, *STRA8*, *Nanos1*, *Nanos2*, *NR5A1*, *Wnt4*
**10**. Chen *et al*., 2010 [7]	?, 1 Male	c.1514T>C, V505A, rs61740566	Hypospadias	-	-	-	Yes	Not located in the conserved site of the protein	-
**11**. Fukami *et al*., 2006 [3]; Fukami *et al*., 2008 [14]	?, 2 male brothers	c.1739A>G, Q580R	Female genitalia	NA	NA	Italian origin. Absent in nephew with same phenotype	Absent in 200 European controls	Transactivation activity on Hes3 equal or higher than WT	-
**12**. Chen *et al*., 2010 [7]	?, 1 male	c.1804C>A, Q602K, rs142908182	Severe hypospadias	-	-	-	No	-	-
**13**. Chen *et al*., 2010 [7]	?, 3 males	c.604ins3Q	Penoscrotal hypospadias, micropenis/chordee	-	-	-	Yes	-	-
**14**. Kalfa *et al*., 2008 [4]	?, 1 male	c.1810ins3Q, 614ins3Q	Isolated coronal hypospadias, intrascrotal testes	NA	NA	-	-	-	-
**15**. Fukami *et al*., 2006 [3]; Fukami *et al*., 2008 [14]	?, 3 patients	c.1985A>G, N662S, rs2073043	DSD	NA	NA	2 Japanese and 1 European origin	In 4 Japanese controls	Transactivation activity on Hes3 similar to WT	-
**15**. Chen *et al*., 2010 [7]	?, 11 males	c.1985A>G, N662S, rs2073043	Hypospadias ranging from perineal to cleaved prepuce	-	-	-	No	Association with hypospadias by screening in a case-control SNP-genotyping study	-
**15**. Kalfa *et al*., 2012 [6]	?	c.1985A>G, N662S, rs2073043	Non-syndromic DSD	-	-	-	-	-	-
**15**. Kalfa *et al*., 2011 [5]	?, males (22/150)	c.1985A>G, N662S, rs2073043	Hypospadias	-	-	-	Yes	Transactivation activity on Hes3 similar to WT	-
**7 + 15**. Kalfa *et al*., 2011 [5]	?, males (16/150)	P359S+N662S	Hypospadias	-	-	-	Yes	Transactivation activity on Hes3 similar to WT	-
**7 + 15**. Kalfa *et al*., 2012 [6]	?, 14 males	P359S+N662S	3 cases: penile posterior hypospadias, cryptorchidism; 5 cases: hypospadias, microphallus; 6 cases: cryptorchidism, microphallus	-	-	-	-	Combined data: incidence of P359S-N662S is higher in DSDs	-
**7 + 15**. Gaspari *et al*., 2011 [12]	?, 1 male	P359S+N662S	Right cryptorchidism, penis 1.5 cm (2.9 mo)	Normal androgen production	-	French with Mediterranian origin	-	Study to evaluate effect of prenatal exposure to environmental endocrine disruptors	N: *AR*, *SRD5A2*, *NR5A1*
**16**. Ruiz-Arana *et al*., 2015 [10]	46,XY, (1/35 DSD ambiguous genitalia), 1 female	c.2030C>T, P677L	Complete gonadal dysgenesis, external female genitalia, no gonads detected, uterus present, primary amenorrhea (13y).	FSH and LH elevated at diagnosis, estrogen low, testosterone normal	-	Mother heterozygous (normal phenotype), father WT. No family history of DSD	No	Abolished transactivation activity on Hes3	N: *SRY*, *NR5A1*, *WT1*
**17**. Fukami *et al*., 2006 [3]; Fukami *et al*., 2008 [14]	?, 1 male	c.2176C>T, R726X rs121909495	Penoscrotal hypospadias, retractile testes, penis 1.2 cm	Normal	Normal	Japanese origin. Mother heterozygous.	Absent in 150 Japanese males	Causes non-mediated RNA decay. Reduced leukocyte transcripts. Transactivation activity on Hes3 similar to WT, protein expression similar to WT	N: *AR*, *SRD5A2*
**18**. Igarashi *et al*., 2015 [9]	46,XY, 1 male (1/180)	c.2041-2A>G, K682fsX1070	Penoscrotal hypospadias, scrotal testes	Normal T, LH and FSH (2y 11 m)	-	-	-	Reduced transactivation activity on Hes3. Reduced mutant protein expression.	-
**19**. Chen *et al*., 2010 [7]	?, male	c.2277C>T, D759D	Hypospadias	-	-	-	No	-	-
**20**. Chen *et al*., 2010 [7]	?, male	c.2284+8A>T	Hypospadias	-	-	-	No	-	-
**21**. Hu *et al*., 1996 [1]; Laporte *et al*., 1997 [2]	1 male, 46,XY	Microdeletion including MAMLD1-MTM1, 5’MAMLD1 gene deletion	Hypospadias, enlarged clitoris/micropenis, bifid scrotum, non- palpable testes. Introitus vagina and vaginal pouch.	Normal 17OHProgesterone, 21DOC, androstenedione, testosterone, dihydrotestosterone, FSH and LH levels	-	-	-	-	-
**22**. Hu *et al*., 1996 [1]; Laporte *et al*., 1997 [2]	1 male, 46,XY	Microdeletion including MAMLD1-MTM1, whole MAMLD1 gene deletion	Perineoscrotal hypospadias. Neuromuscular disorder	-	-	-	-	-	-
**23**. Bartsch *et al*., 1999 [28]	3 males: 2 babies and 1 fetus	Microdeletion including MAMLD1-MTM1, whole MAMLD1 gene deletion	1^st^: abnormal genitalia (undescended right testis and glandular hypospadias) and extreme muscular hypotonia; 2^nd^: bilateral cryptorchidism, penile hypospadias and extreme muscular hypotonia; 3^rd^ (terminated 13 WG): penile hypospadias.	-	-	Mother asthenic, low muscle power and irregular menses	-	-	-
**24**. Tsai *et al*., 2005 [29]	1 male	Microdeletion: deletion MAMLD1ex6-8-MTMR1ex1-2, resulting in a fusion MAMLD1-MTMR1	Without DSD. Myotubular myopathy	-	-	Japanese origin	-	-	-
**25**. Oliveira *et al*., 2013 [30]	1 male	Complex rearrangement including the whole MAMLD1 gene and a fusion 5’MTM1-3’MAMLD1+MTM1	Without DSD. Myotubular myopathy	-	-	-	-	-	-

^a^ the variants were named according to NM_005491.4.

N: normal sequence; NA: not analyzed; ?/-: unknown; GD: gonadal dysgenesis; GOF: gain of function.

To test novel MAMLD1 sequence variations for their disease causing role, we used *Hes3* promoter activation assays as previously performed for other mutations in other laboratories. In addition, we tested for *CYP17A1* promoter activation and an effect on steroid enzyme activity as well as for variable expression of WT and mutant MAMLD1 proteins. Overall, all these studies were not giving conclusive results to explain the role of MAMLD1 gene variants for abnormal sex development. Similar confounding results for functional studies are found in the published literature [Table 3 and [5, 11, 14]]. Although studies in mice revealed a role of MAMLD1 for *Cyp17* gene expression and activity [13], our WT MAMLD1 was only able to modulate the human *CYP17A1* promoter activity, but had no effect on steroid conversion and thus enzyme activity. Human MAMLD1 does not bind to the *Hes3* promoter or to the human *HES3* upstream region directly [14]. Therefore, it has been proposed that MAMLD1 may act through other partners such as the Hes3-DNA-binding transcription factor [14], but to date such partners remain obscure. Our *in silico* search for possible interacting partners of human MAMLD1 was negative when using tools to search for functional partners (string-db.org) or for physical or genetic interactions (http://thebiogrid.org).

To date, twenty-seven *MAMLD1* sequence variations are described, including the 7 novel variants described in this study (Tables 1 and 3). They are all located between exon 4 and intron 6 (Fig 1B) and show the following genotype-phenotype characteristics. Most mutations are only found in DSD patients which present with a broad range of mild to severe phenotype [c325delG (6), S143X [6], E182fsX121 [4], E197X, [3], T202M (present study), L210X and D211N (present study), Q270X, [3], H347Q [6] (present study), P384L [6], Q501Q502 (present study), A503E (present study), Q580R [3], Q602K [7], K682fsX1070 [9], 614ins3Q [4], P677L [10], L724V (present study), R726X, [3], S730S (present study), D759D [7]]. Others are carried by patients and controls [P359S [3, 5, 7] (present study), V505A [4, 7] (present study), 604ins3Q [7], N662S [3, 5–7] (present study)]. MAMLD1 R371X is only described in a control [31]. Finally, in two independent families MAMLD1 mutations [P359S and Q580R [3]] were not found in all 46,XY DSD family members. Thus, genotype-phenotype correlation of MAMLD1 sequence variations and 46,XY DSD is poor.

*MAMLD1* gene variations are also reported in other species, both male and female, and with and without abnormalities in sex development [15–17]. Yet none of them has been clearly related to the DSD phenotype. In dogs, both DSD and controls were carriers [15], in cats only an intronic change has been described [16], and in a male horse with hypospadias a benign mutation was detected, conserved in all *MAMLD1* sequences available at that time [17].

MAMLD1 seems involved in sexual development during fetal life. It is expressed in human fetal testes and ovaries [3, 32], and in mice testes [3, 13]. *Mamld1* is present in mice testes at least from E11.5 which overlaps with the start of androgen biosynthesis (13 dpc) and with the formation of the male external genitalia (16.5 dpc) [13]. *Mamld1* is expressed at low levels in postnatal mice testes until 1 week of age [3, 33]. In human fetal testes, MAMLD1 is expressed at high levels in the second trimester of gestation [3, 32]. In addition, our study shows that it is also expressed in human adult testes and adrenals, but not in fetal adrenal tissue. These results suggest that MAMLD1 may not only have a role during fetal development but also in adult life.

In mice, Mamld1 regulates the expression of steroidogenic enzymes *Cyp17a1*, *Cyp11a1*, *Star*, *Hsd3b1*, *Hsd17b3*, and of genes involved in testes descent (*Amh* and *Insl3*) [13, 20]. It seems involved in testosterone production by regulating Cyp17a1 activity and expression [20]. However, *Mamld1*-KO mice develop normal external genitalia and have normal reproduction, which both depend on testosterone production and Cyp17 activity [13]. Overall, these results challenge the role of MAMLD1 in sex development.

In conclusion, the importance of MAMLD1 in the sexual development becomes less and less clear. Its exact role remains unknown. Studies of MAMLD1 variations in humans suggest that there is a genetic correlation between MAMLD1 sequence variations and DSD. However, the wide range in phenotype and poor genotype-phenotype correlation indicate that MAMLD1 gene variations may not suffice to explain the DSD pathology. Therefore, in DSD patients harboring MAMLD1 sequence variations further genetic studies should be performed searching for additional genetic hits explaining the immense variability. This may be done by specific DSD chips containing a larger array of known genes involved in DSD, which are currently being used in several DSD research projects and should become available soon. Alternatively, an untargeted approach like exome sequencing might be employed that also allows to find novel genes so far unrevealed in DSD [34]. In addition, to find copy number variations array-based comparative genomic hybridization (aCGH) or multiplex ligation-dependent probe amplification (MLPA) may be used [35]. Although so far no multiple hits in DSD genes were described involving MAMLD1, it would not be surprising to find them in the near future with the above mentioned next generation methods.

## Supporting Information

S1 Fig MAMLD1 alignment in mammalian species. Human MAMLD1 (isoform 2, 774 amino acids) was aligned with homologous sequences from 40 mammalian species (length range from 720–820 amino acids). L210 and L724 (1 change each) are conserved along mammalian evolution, followed by S730 (deleted amino acid in 2 species). T202, D211, P359, Q501Q502, A503, V505 and N662 are not conserved, all ranging from 1 to 6 changes. Human changes in positions 202, 347, 505 and 662 present WT in other mammals. The alignments were performed with the CLC Sequence Viewer software (2014 CLC bio, QIAGEN) and show the species common name and the NCBI database (www.ncbi.nlm.nih.gov) accession name/s in brackets. Human: *Homo sapiens*; chimpanzee: *Pan troglodytes*; pygmy chimpanzee: *Pan paniscus*; western lowland gorilla: *Gorilla gorilla gorilla*; crab-eating macaque: *Macaca fascicularis*; pig-tailed macaque: *Macaca nemestrina*; sooty mangabey: *Cercocebus atys*; green monkey: *Chlorocebus sabaeus*; white-tufted-ear marmoset: *Callithrix jacchus*; small-eared galago: *Otolemur garnettii*; Sunda flying lemur: *Galeopterus variegatus*; Chinese tree shrew: *Tupaia chinensis*; Cape golden mole: *Chrysochloris asiatica*; thirteen-lined ground squirrel: *Ictidomys tridecemlineatus*; rabbit: *Oryctolagus cuniculus*; American pika: *Ochotona princeps*; house mouse: *Mus musculus*; prairie deer mouse: *Peromyscus maniculatus bairdii*; Chinese hamster: *Cricetulus griseus*; prairie vole: *Microtus ochrogaster*; Damara mole-rat: *Fukomis damarensis*; Iesser Egyptian jerboa: *Jaculus jaculus*; big brown bat: *Eptesicus fuscus*; horse: *Equus caballus*; Bactrian camel: *Camelus bactrianus*; dog: *Canis lupus familiaris*; alpaca: *Vicugna pacos*; pig: *Sus scrofa*; southern white rhinoceros: *Ceratotherium simum simum*; Pacific walrus: *Odobenus rosmarus divergens*; bottlenosed dolphin: *Tursiops truncatus*; Yangtze River dolphin: *Lipotes vexillifer*; Florida manatee: *Trichechus manatus latirostris*; killer whale: *Orcinus orca*; sperm whale: *Physeter catodon*; sheep: *Ovis aries*; goat: *Capra hircus*; chiru: *Pantholops hodgsonii*; cattle: *Bos taurus*; water buffalo: *Bubalus bubalis*; nine-banded armadillo: *Dasypus novemcinctus*.
(PDF)

S2 Fig Effect of WT and mutant MAMLD1 on CYP17A1 enzyme activity in HEK293 and MA-10 cells. Cells were transiently transfected with MAMLD1 WT and mutant expression vectors. HEK293 cells were also co-transfected with the CYP17A1 expression vector as they do not express it endogenously. The effect of WT and mutant MAMLD1 on CYP17A1 enzyme activity was assessed by measuring the conversion of progesterone (P) to 17-hydroxyprogesterone (17OHP) in non-steroidogenic HEK293 cells, and conversion of P to 17OHP and then to androstenedione (Δ4A) in steroidogenic mouse Leydig MA-10 cells. Steroid production was labeled with [^14^C]progesterone for 60 min. Steroids were extracted and resolved by thin-layer chromatography, then quantified as % conversion. A representative steroid profile obtained from HEK293 (A) and MA-10 (B) cells is shown (n = 2). Similar to experiments performed in NCI-H295R cells (Fig 3B), no effect of MAMLD1 on CYP17-hydroxylase activity was detected. Ve: empty vector; WT: wild type; *: co-transfected with empty vector; NT: non-transfected.
(TIF)

S3 Fig Protein expression of MAMLD1. Picture of the original uncropped and unadjusted Western blots for myc-MAMLD1 (A) and actin (B) corresponding to Fig 4. Ve: empty vector; WT: wild type; NT: non-transfected.
(TIF)

S1 File Relevant experimental data. Data corresponding to the transactivation studies are provided, which include: raw data from the luciferase assays, values behind statistics and original graphs.
(XLSX)

S1 Table Primers used in this study. (DOCX)
