# Supplementary material for: Human MAMLD1 Gene Variations Seem Not Sufficient to Explain a 46,XY DSD Phenotype
Source: PLoS One. 2015 Nov 16;10(11):e0142831. doi: 10.1371/journal.pone.0142831 (PMC4646284; doi:10.1371/journal.pone.0142831)
Supplement: S1 Fig — Human MAMLD1 (isoform 2, 774 amino acids) was aligned with homologous sequences from 40 mammalian species (length range from 720–820 amino acids). L210 and L724 (1 change each) are conserved along mammalian evolution, followed by S730 (deleted amino acid in 2 species). T202, D211, P359, Q501Q502, A503, V505 and N662 are not conserved, all ranging from 1 to 6 changes. Human changes in positions 202, 347, 505 and 662 present WT in other mammals. The alignments were performed with the CLC Sequence Viewer software (2014 CLC bio, QIAGEN) and show the species common name and the NCBI database (www.ncbi.nlm.nih.gov) accession name/s in brackets. Human: Homo sapiens; chimpanzee: Pan troglodytes; pygmy chimpanzee: Pan paniscus; western lowland gorilla: Gorilla gorilla gorilla; crab-eating macaque: Macaca fascicularis; pig-tailed macaque: Macaca nemestrina; sooty mangabey: Cercocebus atys; green monkey: Chlorocebus sabaeus; white-tufted-ear marmoset: Callithrix jacchus; small-eared galago: Otolemur garnettii; Sunda flying lemur: Galeopterus variegatus; Chinese tree shrew: Tupaia chinensis; Cape golden mole: Chrysochloris asiatica; thirteen-lined ground squirrel: Ictidomys tridecemlineatus; rabbit: Oryctolagus cuniculus; American pika: Ochotona princeps; house mouse: Mus musculus; prairie deer mouse: Peromyscus maniculatus bairdii; Chinese hamster: Cricetulus griseus; prairie vole: Microtus ochrogaster; Damara mole-rat: Fukomis damarensis; Iesser Egyptian jerboa: Jaculus jaculus; big brown bat: Eptesicus fuscus; horse: Equus caballus; Bactrian camel: Camelus bactrianus; dog: Canis lupus familiaris; alpaca: Vicugna pacos; pig: Sus scrofa; southern white rhinoceros: Ceratotherium simum simum; Pacific walrus: Odobenus rosmarus divergens; bottlenosed dolphin: Tursiops truncatus; Yangtze River dolphin: Lipotes vexillifer; Florida manatee: Trichechus manatus latirostris; killer whale: Orcinus orca; sperm whale: Physeter catodon; sheep: Ovis aries; goat: Capra hircus; chiru: Pantholo [file pone.0142831.s001.pdf]

Supplemental Figure 1

human (NP_005482 XP_931544 XP_942829)	MDDWKSRLVI	KSMLPHFAMV	GNRQEPRKLQ	ESGKKPSWME	EE-DLSFLYK	SSPGRKHQGT	VKRRQEE-DH	FQ-FPDMADG	77
chimpanzee (XP_009438041)	MDDWKSRLVI	KSMLPHFAMV	GNRQEPRKLQ	ESGKKPSWME	EE-DLSFLYK	SSPGRKHQGT	VKRRQEE-DH	FQ-FPDMADG	77
pygmy chimpanzee (XP_008966316)	MDDWKSRLVI	KSMLPHFAMV	GNRQEPRKLQ	ESGKKPSWME	EE-DLSFLYK	SSPGRKHQGT	VKRRQEE-DH	FQ-FPDMADG	77
western lowland gorilla (XP_004065053)	MDDWKSRLVI	KSMLPHFAMV	GNRQEPRKLQ	ESGKKPSWME	EE-DLSFLYK	SSPGRKPQGT	VKRRQEE-DH	FQ-FPDMADG	77
crab-eating macaque (XP_005594862)	MDDWKSRLVI	KSMLPHFAMV	GNRQEPRKLQ	ESGKKPSWME	EE-DLSFLYK	SSPGRKHQGT	VKRRQEE-DH	FQ-FPDMADG	77
pig-tailed macaque (XP_011764131)	MDDWKSRLVI	KSMLPHFAMV	GNRQEPRKLQ	ESGKKPSWME	EE-DLSFLYK	SSPGRKHQGT	VKRRQEE-DH	FQ-FPDMADG	77
sooty mangabey (XP_011936741)	MDDWKSRLVI	KSMLPHFAMV	GNRQEPRKLQ	ESGKKPSWME	EE-DLSFLYK	SSPGRKHQGT	VKRRQEE-DH	FQ-FPDMADG	77
green monkey (XP_007991156)	MDDWKSRLVI	KSMLPHFAMV	GNRQEPRKLQ	ESGRKPSWME	EE-DLSFLYK	SSPGRKHQGT	VKRRQEE-DH	FQ-FPDMADG	77
white-tufted-ear marmoset (XP_008988264)	MDDWKSRLVI	KSMLPHFAMV	GNRQEPRKLQ	ESGKKPSWMD	EEGNLSFLYK	SSPGRKHQGT	VKRRXEE-DH	FQ-FPDMADG	78
small-eared galago (XP_003800989)	MDDWKSRLVI	KSMLPHFTMV	GNRQEPRKLQ	ESR-------	----------	---------T	IKRRQEG-DN	FQ-FPGMADG	52
Sunda flying lemur (XP_008574565)	MDDWKNRLVI	KSMLPHFAMV	GNRQEPRKLQ	ES--------	----------	------PQGT	IKRRQEG-DN	FQ-FPGMADG	54
Chinese tree shrew (XP_006171265)	MDDWKNRLVI	KSVLPRFTAV	GNRQESRKLQ	ES--------	----------	--------GT	LKRRQEG-ES	FQ-FTGMADS	52
Cape golden mole (XP_006874840)	MDEWKSRLVI	KSSLPHYAMV	GNRQEPRKLQ	ES--------	----------	------PQGT	TKRRQAG-DN	FQQYPVMADG	55
hirteen-lined ground squirrel (XP_005338579)	MDDWKSRLVI	KSMLPHFAMV	GNRQEPRKLQ	ES--------	----------	------PQGT	LKRRQEG-DN	YQ-FAGMADG	54
rabbit (XP_008273259)	MDDWKSRLVI	KSMLPHFAMV	GNRQEPRKLQ	ESS-------	----------	-------QGA	IKRRQE-RDH	FQ-FPGMADG	54
American pika (XP_004598606)	MDDWKSRLII	KSMLPHFAVV	GNRQEPRKLQ	ES--------	----------	--------GT	IKRRQE--EH	F-LFPGMADG	51
house mouse (NP_001074823 XP_903797)	MDDWKSRLVI	ENMLPHFNMV	GNRQETRKLQ	ES--------	----------	--------GT	SKRRQEG-EN	FH-FTGMADG	52
prairie deer mouse (XP_006973990)	MDDWKSRLVI	ESMLPHFNMV	GNRQEPRKLQ	ES--------	----------	--------GT	SKRRQEG-DN	FH-FTGMADG	52
Chinese hamster (XP_007629791)	MDDWKSRLVI	ESMLPHFNMV	GNRQEPRKLQ	ES--------	----------	--------GT	SKRRQEG-DN	FH-FAGMADG	52
prairie vole (XP_005369242)	MDDWKSRLIL	ESMLPHFNMA	GNRQEPRRLQ	ES--------	----------	--------GT	SKRRQEG-DN	FH-FTGMADG	52
Damara mole-rat (XP_010637336)	MDDWKSRLVV	KSMLPHFAMV	GNRQEPRKLQ	ESS-------	----------	-------QGT	NKRRQEE-DD	FQ-FPGMADG	54
lesser Egyptian jerboa (XP_004668990)	MDDWKSRLVI	ESMLPHFTMV	GNRQEPRKLQ	ES--------	----------	--------GT	TKRRQEGGDN	FQ-FAGMADG	53
big brown bat (XP_008156940)	MDDWKTRLVI	KSMLPHIAMV	GNRQEPRKLQ	ES--------	----------	------PQGT	IKRRQEG-DN	FQ-FPGMADG	54
horse (NP_001166929 XP_001505137)	MDDWKSRLVI	KSMLPHFTVV	GNRQEPRKLQ	ES--------	----------	------PQGT	LKRRQEG-DN	FQ-FPGMADG	54
Bactrian camel (XP_010954813)	MDDWKSRLVV	KSMLPHFTMV	GDRQEPRKLQ	ES--------	----------	------PQGT	IKRRQEG-DN	FQ-LSGMADG	54
dog (XP_005641990)	MDDWKSRLVI	KSMVPHYAVV	GNRQEPRKLQ	ES--------	----------	--------GT	FKRRQEA-DN	FQ-FPGMADG	52
alpaca (XP_006216256)	MDDWKSRLVI	KSMLPHFTMV	GNRQEPRKLQ	ESNRKPGWVE	EENS-SFLCR	SSPGGKPQGT	IKRRQEG-DN	FQ-LSGMADG	77
pig (XP_003135512)	MDDWKSRLVI	KSMLPHFAMV	GNRQEPRKLQ	ES--------	----------	--------GT	IKRRQEG-DN	FQ-LSGMADG	52
southern white rhinoceros (XP_004443252)	MDDWKSRLVI	KSMLPHFAMV	GNRQEPRKLQ	ES--------	----------	--------GT	IKRRQEG-DN	FQ-FPGMADG	52
Pacific walrus (XP_004407178)	MDDWKSRLVI	KSMVPHFAMV	GNRQEPRKLQ	ES--------	----------	--------GT	IKRRQEG-DN	FQ-FPGMADG	52
bottlenosed dolphin (XP_004325294)	MDDWKSRLVI	KSMLPHFAMV	GNRQEPRKLQ	ESNRKPFWFE	QE-DLSHFTK	QSPGRSEKGT	IKRRQEG-ET	FQ-LSGMAEG	77
Yangtze River dolphin (XP_007452175)	MDDWKNRLVI	KSALPHLAMA	GNRQEPRRLQ	ES--------	----------	------PQGT	IKRRQEG-ET	FQ-LSGMAEG	54
Florida manatee (XP_004390770)	MDDWKSRLVI	KSMLPHYAMV	GNRQEPRKLQ	ES--------	----------	------PQGT	VKRRQAG-DS	FQHFPGMADG	55
killer whale (XP_004284138)	MDDWKSRLVI	KSMLPHFAMV	GNRQEPRKLQ	ESNRKPFWFE	QE-DLSHFTK	QSPGRSEKGT	IKRRQEG-ET	FQ-LSGMAEG	77
sperm whale (XP_007129626)	MDDWKSRLVI	KSMLPHFAMV	GNRQEPRKLQ	ES--------	----------	------PQGT	IKRRQEG-ET	FQ-LSGMAEG	54
sheep (XP_011962574)	MDDWKSRPVV	KSMLPHFAVV	GNRQEPRKLQ	ESA-------	----------	-------QGT	TKRRQEGED-	FQ-LSGMGDE	54
goat (XP_005700529)	MDDWKSRLVV	KSMLPHFAVV	GNRQEPRKLQ	ES--------	----------	--------GT	TKRRQEGED-	FQ-LSGMGDE	52
chiru (XP_005962515)	MDDWKSRLVV	KSMLPHFTVV	GNRQEPRKLQ	ES--------	----------	--------GT	TKRRQEGED-	FQ-LSGMGDE	52
cattle (XP_010798654)	MDDWKSRLVV	KSMLPHFAMV	GNRQEPRKLQ	ESA-------	----------	-------QGT	TKRRQEGED-	FQ-LSGMGDE	54
water buffalo (XP_006059754)	MDDWKSRHVV	KSMLPHFAMV	GNRQEPRKLQ	ESA-------	----------	-------QGT	TKRRQEGED-	FQ-LSGMGDE	54
nine-banded armadillo (XP_004448017)	MDDWKSRLVI	KSTLPHFATV	GNRQEPRKLQ	ES--------	----------	------PQGT	IKRRQEG-DN	FQ-FPGMAEG	54

Supplemental Figure 1

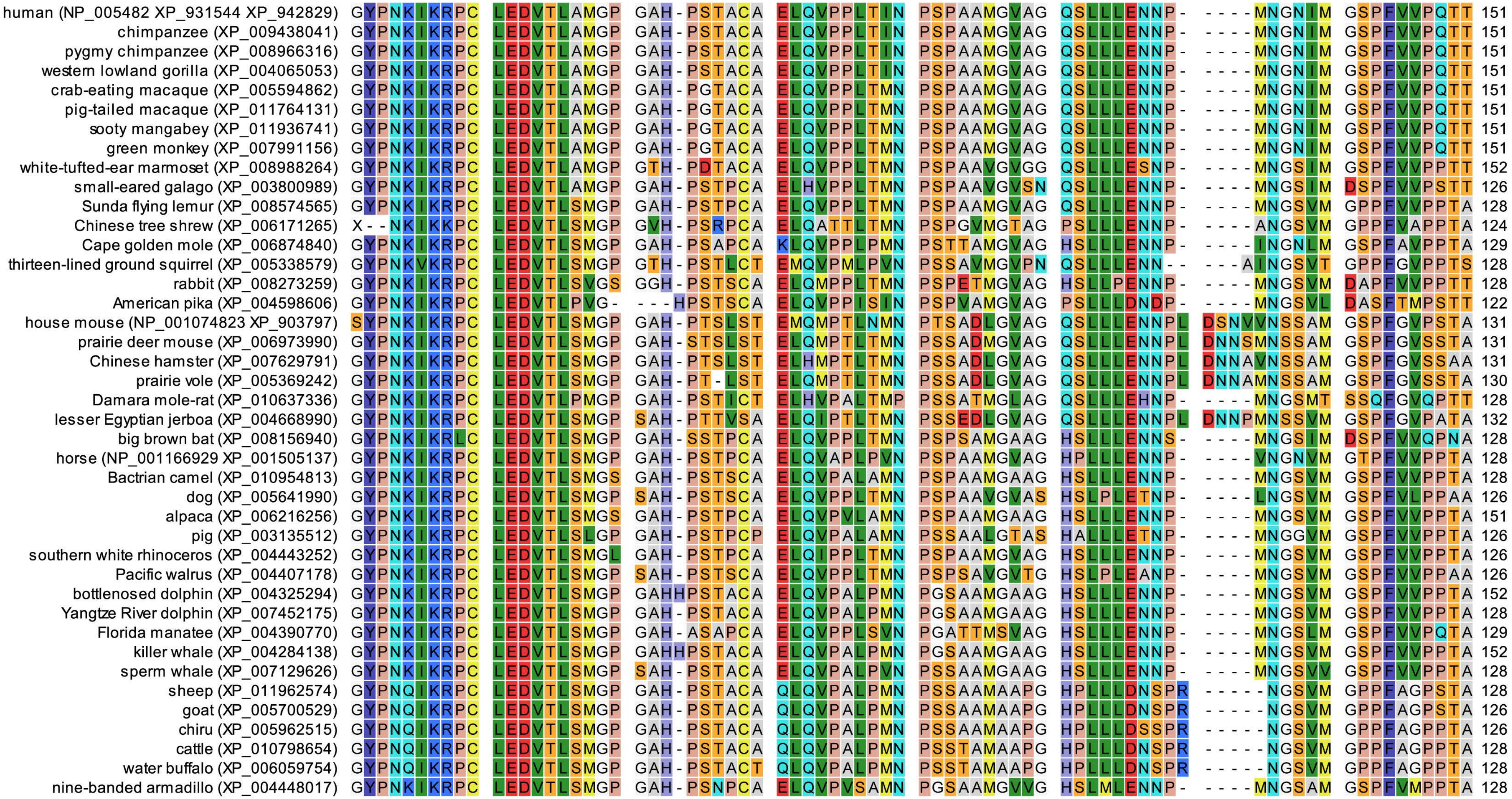

Supplemental Figure 1

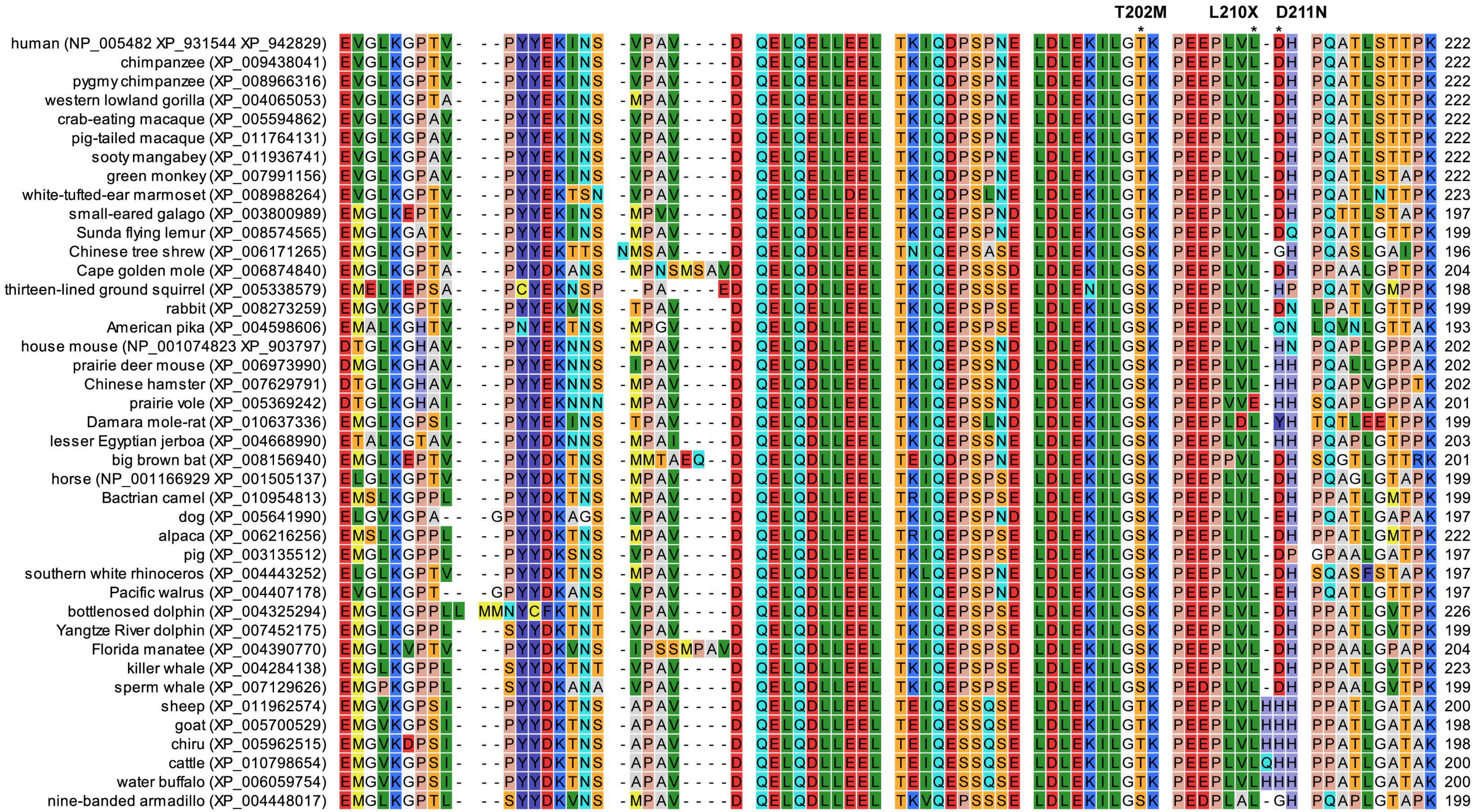

Supplemental Figure 1

```
human (NP_005482 XP_931544 XP_942829)        P-SVQMSHLE SLASSKEFAS SCSQVTGM-- SLQI-PSSST GISYSIPSTS KQIVSPSSSM A--------- ---QSKSQVQ 286
chimpanzee (XP_009438041)                    P-SVQMSHLE SLASSKEFAS SCSQVTGM-- SLQI-PSSST GISYSIPSTS KQIVSPSSSM A--------- ---QSKSQVQ 286
pygmy chimpanzee (XP_008966316)              P-SVQMSHLE SLASSKEFAS SCSQVTGM-- SLQI-PSSST GISYSIPSTS KQIVSPSSSM A--------- ---QSKSQVQ 286
western lowland gorilla (XP_004065053)       P-SVQMSHLE SLASSKEFAS SCSQVTGM-- SLQI-PSSST GISYSIPSTS KQIVSPSSSM A--------- ---QSKSQVQ 286
crab-eating macaque (XP_005594862)           P-SVQMSHLE SLASSKEFAS SCSHVTGM-- SLQI-PSS-T GISYSIPSTS KQIVSPSSSM A--------- ---QSKSQVQ 285
pig-tailed macaque (XP_011764131)            P-SVQMSHLE SLASSKEFAS SCSHVTGM-- SLQI-PSS-T GISYSIPSTS KQIVSPSSSM A--------- ---QSKSQVQ 285
sooty mangabey (XP_011936741)                P-SVQMSHLE SLASSKEFAS SCSHVTGM-- SLQI-PSS-T GISYSIPSTS KQIVSPSSSM A--------- ---QSKSQVQ 285
green monkey (XP_007991156)                  P-SVQMSPLE SLASSKEFAS SCSQVTGM-- SLQI-PSS-T GISYSIPSTS KQIVSPSSSM A--------- ---QSKSQVQ 285
white-tufted-ear marmoset (XP_008988264)     P-SVQMSHLE SLASSKDFAS SCSQVTGV-- SLQI-PPSCT GISYSIPSTS KQMVSQSSSM A--------- ---QSKSQVQ 287
small-eared galago (XP_003800989)            P-SVQMPHLE GLGSGKEFAS SCSQVTGV-- SLQTHPPSSA GISYSIPSTS KQMVSPSSST A--------- ---QAKNQVQ 262
Sunda flying lemur (XP_008574565)            P-SGQMPHLE SLGSSKEFAS SCSQVTGM-- SLQI-PPSST GISYSIPSTS KQIVSPSSST A--------- ---QAKNQVQ 263
Chinese tree shrew (XP_006171265)            PL-VQMPQLE GPSSSKEFAS SCSQVPGV-- SLQT-PASST GISYSVPSTN KQMVSPTSST S--------- ---QTKNQAQ 260
Cape golden mole (XP_006874840)              PP-VHMPRLE GLGSSKDFAS SCSQVAGV-- SLHI-PLSSA GMNYTIPSTS KQLVSPSSST A--------- ---QAKNQGQ 268
thirteen-lined ground squirrel (XP_005338579) LP-VQMPQME SVCSSKAFAS SCSQVTGV-- SVPIMP-SST GISHSVPSTG KQMVSVSPST T--------- ---QAKNQAQ 262
rabbit (XP_008273259)                        ISVHHMPHME SLGSSKEFAS SCSQVTGAPF PI---PPSST GVSYSIPSSR KQMVSPSSST A--------- ---QAKNQGQ 264
American pika (XP_004598606)                 PTVHHMAHLE SLGSSKDFAS SCSQVSST-- SLPM-PLSST DLSYSIPSTS KHMISPSSST A--------- ---ETKNQVQ 258
house mouse (NP_001074823 XP_903797)         LP-VQMPHME SLGSSKEFAS SCSQVAGT-- SLPIMP-SST GMSYSIPSSS KQIVSSSSST AQA------- ---QVKNQVQ 268
prairie deer mouse (XP_006973990)            PP-VQTPHME NLGPSKDYAS SCSQVTGT-- SLPIMP-SST GISYSIPSTS KQIVSPSSST AQA------- ---QVKNQVQ 268
Chinese hamster (XP_007629791)               LP-VQMPHME SLGSSKEFAS SCSQVTGT-- SLPILP-SST GISYSIPSTS KQIVSPSSST AQA------- ---QVKNQAQ 268
prairie vole (XP_005369242)                  LP-VQMPHME SLGPSKEYAS SCSQATDT-- SLPVLP-SST EINYPIPPTS KQIASPSSST AQA------- ---QVKNQAQ 267
Damara mole-rat (XP_010637336)               LP-VQVPQLE DLDSGKEFTS SCSQVTGV-- SLPILP-SST GISYSVPSTS KQMASPSSSI A--------- ---QAKNQ-- 261
lesser Egyptian jerboa (XP_004668990)        L---QMPHLE SLG------S SCSQVTGT-- SLPILP-SST GISYSIPSTS KQMVSPSSST AQA------- ---QAKNQA- 260
big brown bat (XP_008156940)                 A-SAQMPHLE NLGSSKEFAS GCSQVTGV-- SLQI-PPSSA GISYAIPSTS KQMVTPSSAI T--------- ---QAKSQVQ 265
horse (NP_001166929 XP_001505137)            P-SAQMPHLE SLASGKEFAS SCSQVPGV-- SLQI-PPSSA GVSYAIPSTS KQLVSPSSST A--------- ---QAKNQVQ 263
Bactrian camel (XP_010954813)                PPSVQMPPLE SLGSSKEFAS SCSQVTSV-- SLQI-PPSSA GISYVIPATG KQMVSPSSLG T--------- ---QAKKQAQ 264
dog (XP_005641990)                           P-SAAIPHLE SLATSKEFTS SCSQLPGAAT SLHV-PPSPG GLNYVIPSAS KQVASPGSSA AAAAAAAAAA AASQAKSQVQ 275
alpaca (XP_006216256)                        PPSVQMPPLE SLGCSKEFAS SCSQVTGV-- SLQI-PPSSA GISYVIPATG KQMVSPSSLG T--------- ---QAKKQAQ 287
pig (XP_003135512)                           P-AVQMPPLE SLASGKDFAS SCSQVTGV-- SLPI-PPSSV GVSYGVPSTS KQMASPSSST A--------- ---QAQSQVP 261
southern white rhinoceros (XP_004443252)     P-SVQMPHLE SLGSSKEFAS SCSQVTGV-- SLQI-PPSSA GISYAIPSTS KQLVSPSSST A--------- ---QAKNQVQ 261
Pacific walrus (XP_004407178)                P-PVQMPHLE SLGPGKEFAS SCSQLPGA-- SLQV-PTPPA GISYVIPSTS KQVASPSSTA A--------- ---QAKSQVQ 261
bottlenosed dolphin (XP_004325294)           P-AVQMPHLE SLGSSKDFAS SCSQVPGV-- SLQI-PPSSA GISYAIPSTS KQMVSPSSST A--------- ---QAKSQVQ 290
Yangtze River dolphin (XP_007452175)         P-AVQMPHLE SLGSSKDFAS SCSQVPGV-- SLQI-PPSSV GISYVIPSTS KQMVSPSSST S--------- ---QAKSQAQ 263
Florida manatee (XP_004390770)               PP-VHMPHSE GLGSTKEFAS SCSQVAGV-- SLHI-APSSA GISYTLPASS KQMASPSSPT A--------- ---QAKSQGR 268
killer whale (XP_004284138)                  P-AVQMPHLE SLGSSKDFAS SCSQVPGV-- SLQI-PPSSA GISYVIPSTS KQMVSPSSST A--------- ---QAKSQVQ 287
sperm whale (XP_007129626)                   P-AVQMPHLE SLGSGKDFAS SCGQVPGV-- SLQI-PPSSA GLSYVIPSTS KQMVSPSSST A--------- ---RAKSQ-- 261
sheep (XP_011962574)                         P-TVQMPRLE SLSSGKDFAS SCSQVTGV-- SLQI-PPSSA GLSYAITPAS KQMASPSSST V--------- ---QAKNQAQ 264
goat (XP_005700529)                          P-TVQMPRLE SLSSDKDFAS SCSQVTDV-- SLQI-PPSSA GLSYAITPAS KQMASPSSST V--------- ---QAKNQAQ 262
chiru (XP_005962515)                         P-TVQMPRLE SLSSGKDFAS SCSQVTGV-- SLQI-PPSSA GLSYAITPAN KQMASPSSST V--------- ---QAKNQAQ 262
cattle (XP_010798654)                        P-TVQMPRLE SLSSSKDFAS SCSLDTGV-- SLQI-PPSSA GFSYAITPAS KQMASPSSST G--------- ---QAKNQAQ 264
water buffalo (XP_006059754)                 P-TVQMPRLE SLSSSKDFAS SCSQVTGV-- SLQI-PPSSA GLSYAITPAS KQMASPSSST V--------- ---QAKNQAQ 264
nine-banded armadillo (XP_004448017)         PP-VQMPHLE SLAPSKEFAS SCSQAVGVP- -LQI-PASSA GISYALPSTS KQMVLPSSSM A--------- ---QAKSQGP 263
```

Supplemental Figure 1

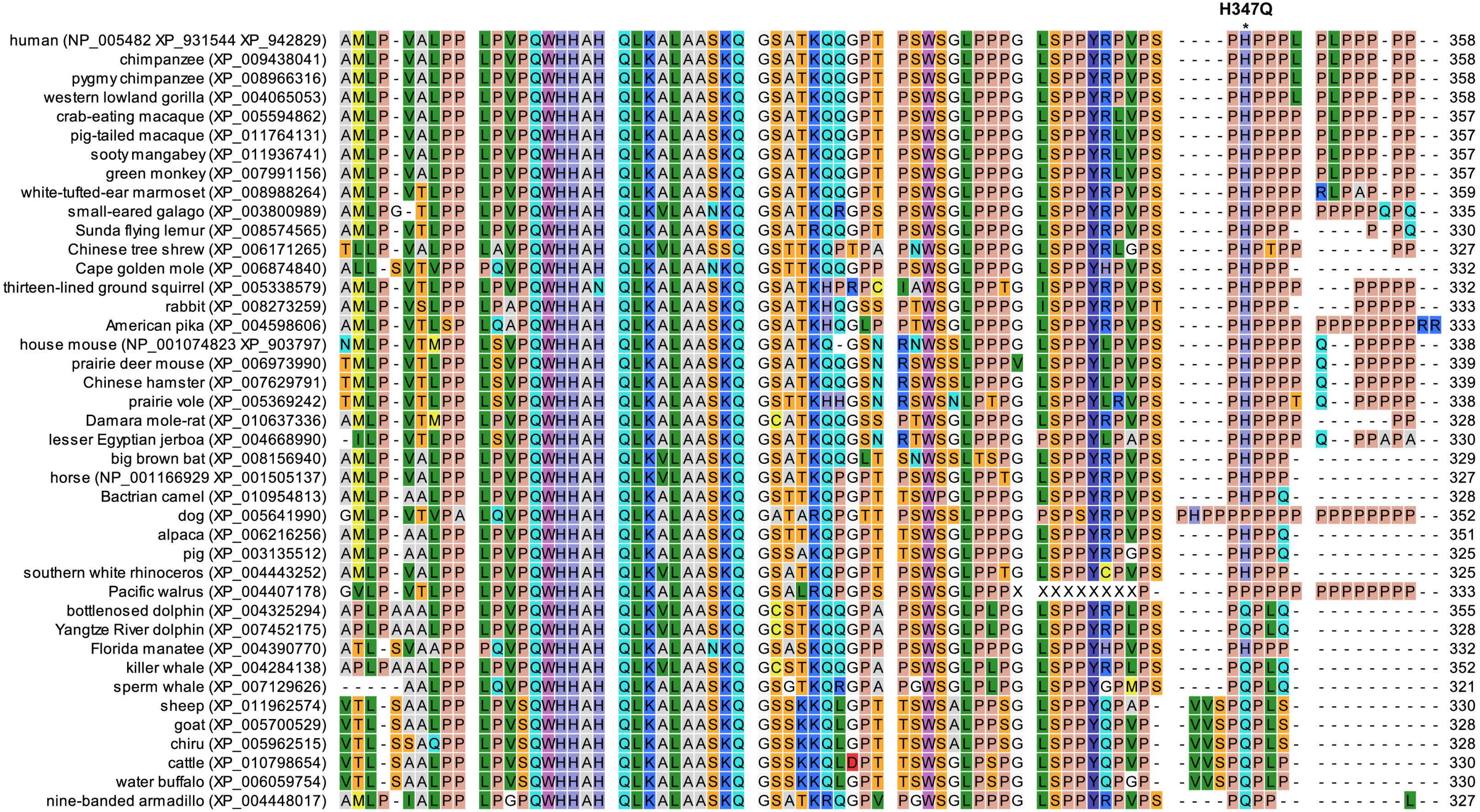

Supplemental Figure 1

P359S
*

human (NP_005482 XP_931544 XP_942829)	PFSPQSLMVS	CMSSNTLSGS	TLRGSPNALL	SSMTSSSNAA	LGPAMPYAPE	KLPSPALTQQ	PQFGPQSSIL	ANLMSSTIKT	438
chimpanzee (XP_009438041)	PFSPQSLMVS	CMSSNTLSGS	TLRGSPNALL	SSMTSSSNAA	LGPAMPYAPE	KLPSPALTQQ	PQFSPQSSIL	ANLMSSTIKT	438
pygmy chimpanzee (XP_008966316)	PFSPQSLMVS	CMSSNTLSGS	TLRGSPNALL	SSMTSSSNAA	LGPAMPYAPE	KLPSPALTQQ	PQFSPQSSIL	ANLMSSTIKT	438
western lowland gorilla (XP_004065053)	PFSPQSLMVS	CMSSNTLSGS	TLRGSPNALL	SSMTSSSNAA	LGPAMPYAPE	KLPSPALTQQ	PQFGPQSSIL	ANLMSSTIKT	438
crab-eating macaque (XP_005594862)	PFSPQSLMVS	CMSSNTLSGS	TLRGSPNALL	SSMTSSSNAA	LGPTMPYAPE	KLPSPALTQQ	PQFGPQSSLL	ANLVSSTIKT	437
pig-tailed macaque (XP_011764131)	PFSPQSLMVS	CMSSNTLSGS	TLRGSPNALL	SSMTSSSNAA	LGPTMPYAPE	KLPSPALTQQ	PQFGPQSSLL	ANLVSSTIKT	437
sooty mangabey (XP_011936741)	PFSPQSLMVS	CMSSNTLSGS	TLRGSPNALL	SSMTSSSNAA	LGPTMPYAPE	KLPSPALTQQ	PQFGPQSSLL	ANLVSSTIKT	437
green monkey (XP_007991156)	PFSPQSLMVS	CMSSNTLSGS	TLRGSPNALL	SSMTSSSNAA	LGPTMPYAPE	KLPSPALTQQ	PQFGPQSSLL	ANLVSSTIKT	437
white-tufted-ear marmoset (XP_008988264)	PFSPQSLMVS	CMSSNTLSGS	TLQGSPNALL	SSMTSSSNAA	LGPAMPYAPE	KLPSPALTQQ	PQFGPQSSLL	ANLVSSTIKT	439
small-eared galago (XP_003800989)	PFSTQSLMVS	CMSSSSLSGS	TLRGSPNALL	SSMAPSSSAA	LGPAMAYAPE	KLPSPTLTRQ	PQFSPQSSIL	ANLVSSTIKN	415
Sunda flying lemur (XP_008574565)	PFSPQSLMVS	CMSSNNLSGN	TMQGSANALL	SSMVSSSNAA	LGPAMPYASE	KLPSPALSQQ	PQFSPQSPIL	ANLVSSTIKS	410
Chinese tree shrew (XP_006171265)	PFSPSSLMVS	CMSPSSLPSS	AFQGAPNALL	SSMASSSSAS	LGPAAPYAPE	KPSSSALNQQ	PQFSPQSSIL	ANLVSSSVKS	407
Cape golden mole (XP_006874840)	PFSPQSLMVS	CMSSSNLPGS	CLQGSPNALL	AGMATNSNPA	LGPVMPYVPE	KLPSPALSQQ	SQFSPQSSIL	ANLVSSNIKN	412
thirteen-lined ground squirrel (XP_005338579)	PFGSQSFMGP	CISSNSSSGS	TVQASPNALL	SSMASGSSAT	MGPTLHYVPE	KHPSVAVNQQ	PQFGQQGSIL	ANLVSSAIKS	412
rabbit (XP_008273259)	AFSPQSLMVS	CMAPHSLPGS	SLQGSPNALL	SSMAPNSSGA	LGPALPYATE	KLPSTALSQQ	SQFSPQSSIL	ANLVSSTIKS	413
American pika (XP_004598606)	TFSPQSLAVS	CMAPNSLQGS	SLQGSPNALL	SSMVPSSSAT	LGRPMPYTAE	KLPSPSLNQQ	QQFTPQSSIL	ANLVRSTIKS	413
house mouse (NP_001074823 XP_903797)	-FSPQNFTAS	CMSSSSLSGS	AVQSSPNALL	SSMAPSSNAS	LGPTLPYVPA	KLPGLPLNQQ	PQFSPQSSIL	ANLVSSSVKS	417
prairie deer mouse (XP_006973990)	-FSPQNFTAS	CMSSNSLSGS	AVQSSPNALL	SSMAPSSNAA	LGPTLPYAPA	KLTGLPHNQQ	PQFSPQSSIL	ANLVSSTVKS	418
Chinese hamster (XP_007629791)	-FSPQNFTAS	CMSSNNLSGS	VVQSSPNALL	SSMAPSSNAA	LGPSLPYAQA	KHPGLPHNQQ	PQFSPQSSIL	ANLVSSTVKS	418
prairie vole (XP_005369242)	PFSPQNFTAP	CMSSNSLSGS	AAQSSSNALL	SSMAPSSNAA	LGPTLPYAPA	KLPALPHNQK	PQFSPQSSIL	ANLVSSSVKS	418
Damara mole-rat (XP_010637336)	PFSPQSFMVS	CMSSSSSSSS	TLQGSPNDLL	SSMASGSSAN	LGPTLPYAPE	KLPSPALSQQ	PQFSPQSSIL	ANLVSSTIKS	408
lesser Egyptian jerboa (XP_004668990)	-FSPHNFPAS	CMSSNSLSGS	TVQGSSSALL	SSMAPSSSAS	LGPTLPYAPA	KLPGLGLNQQ	PQFSPQGSIL	ANLVSSTVKS	409
big brown bat (XP_008156940)	PFSPQSLMVS	CMSSSNLPGS	TLQGSPNALL	SSMASSSNAA	LGPTMSYAPE	KLPSPSLNQQ	PQFSPQSSIL	ANLVSSTMKS	409
horse (NP_001166929 XP_001505137)	PFSPQSLMVS	CMSSSNLPGS	TLQGSPNALL	SSMVSGSSAA	LGPAMPYAPE	KLPSPALSQQ	PQFSPQNSIL	ANLVSSTIKN	407
Bactrian camel (XP_010954813)	PFSSPSLMVS	CMSSSSLPGS	ALQGSPNALL	SSVAPSSSTA	LGPTLPYAPE	KLPSPALHQQ	PQFSPQSSIL	ANLVSSTIKN	408
dog (XP_005641990)	QFSPQSLMVS	CMASSNLPGS	SFQASPNALL	ASMASASSAG	LGPPMLYTPD	KLPSPALSQQ	PPFSPQSSIL	ANLVSSGVKS	432
alpaca (XP_006216256)	PFSSPSLMVS	CMSSSSLPGS	ALQGSPNALL	SSVAPSSSTA	LGPTLPYAPE	KLPSPALHQQ	PQFSPQSSIL	ANLVSSTVKN	431
pig (XP_003135512)	PFSPQGLMGS	CMSSSSLPGS	TLHGSPGALL	SSGAPSSSSA	LGPALPYAPE	KLPSPALHQQ	PQFSPQNSIL	AHLVSSGIKT	405
southern white rhinoceros (XP_004443252)	PFSPQSLMVS	CMSSSNLPGS	TLQGSPNALL	SSMASSSNAA	LGPTMPYAPE	KLPSPALNQQ	PQFSPQNSIL	ANLVSSTIKN	405
Pacific walrus (XP_004407178)	QFSPQSLMVS	CMASSNLPGS	AFQASPNALL	ASMASGGSAA	LGPAMLYTPD	KLPGPALSQQ	PQFSPQSSLL	ANLVSSGVKS	413
bottlenosed dolphin (XP_004325294)	PFSPQGLMVS	CMSSS-----	ALQGSPNALL	SSVAPSSGAA	LGPAMTYAPE	KLPSPALQQQ	PQFSPQPSIL	ANLVSSTIKQ	430
Yangtze River dolphin (XP_007452175)	PFSPQGLMVS	CMSSSSLPAS	ALQGSPNALL	SSVAPSSGAA	LGPAMTYAPE	KLPSPALQQQ	PQFSPQPSIL	ANLVSSTIKQ	408
Florida manatee (XP_004390770)	PFSPQSLMVS	CMSSSNLLGS	SLQGSPNALL	AGMATSSNAA	LGPVMPYVSE	KLPSPALNQQ	SQFSPQSSIL	ANLVSSNIKN	412
killer whale (XP_004284138)	PFSPQGLMVS	CMSSSSLPAS	ALQGSPNALL	SSVAPSSGAA	LGPAMTYAPE	KLPSPALQQQ	PQFSPQPSIL	ANLVSSTIKQ	432
sperm whale (XP_007129626)	PFSPQGLMVS	CMSSSSLPAS	ALQGSPNALP	SSVAPSSGAA	LGPAVTYAPE	RLPSPALQQQ	PPYSPQPSVL	ANLVSSTIKH	401
sheep (XP_011962574)	PFSPQGLMVS	CMSSNSLPGS	TLQGSPNALL	SSLAPSSSTV	LGPARPYVPE	KFPSPTLQQP	PPFSAQTSIL	ANLMSSTIKH	410
goat (XP_005700529)	PFSPQGLMVS	CMSSNSLPGS	TLQGSPNALL	SSLAPSSSTV	LGPARPYVPE	KFPSPTLQQP	PPFSAQTSIL	ANLVSSSIKH	408
chiru (XP_005962515)	PFSPQGLMVS	CMSSNSLPGS	TLQGSPNALL	SSLAPSSSTV	LGPARPYVPE	KFPSPTLQQP	PPFSAQTSIL	ANLVSSTIKH	408
cattle (XP_010798654)	PFSPQGLMVS	CMSSSSLPGT	TLQGSPNTLL	SSLAPSSSTV	LGPARPYAPE	KFPSPTLQQP	PPFSTQTSIL	ANLMSSTVKH	410
water buffalo (XP_006059754)	PFSPQGLMVS	CMSSSSLPGT	TLQGSPNALL	SSLAPSSSTV	LGPARPYAPE	KFPSPTLQQS	PPFSTQTSIL	ANLMSSTVKH	410
nine-banded armadillo (XP_004448017)	PFSQQSLMVS	CMSSSSL-GS	TLQGLPNALL	SSMATSSSAA	LGTPVPYAPE	KPPSAALHQQ	PPFGPQSSIL	ANLMSSTIKT	406

Supplemental Figure 1

human (NP_005482 XP_931544 XP_942829)	PQGH-LMSAL	PASNPGPSPP	YRPEKLSSPG	LPQQSFTPQC	SLIRSLTPTS	-NLLS-----	----------	------QQQQ	495
chimpanzee (XP_009438041)	PQGH-LMSAL	PASNPGPSPP	YRPEKLSSPG	LPQQSFTPQC	SLIRSLTPTS	-NLLS-----	----------	------QQQQ	495
pygmy chimpanzee (XP_008966316)	PQGH-LMSAL	PASNPGPSPP	YRPEKLSSPG	LPQQSFTPQC	SLIRSLTPTS	-NLLS-----	----------	------QQQQ	495
western lowland gorilla (XP_004065053)	PQGH-LMSAL	PASNPGPSPP	YRPEKLSSPG	LPQQSFTPQC	SLIRSLTPTS	-NLLSQQQQQ	QQ----QQQQ	QQQQQQQQQQ	512
crab-eating macaque (XP_005594862)	PQGH-LMSAL	PASNPGPSPP	YRPEKLSSPG	LPQQSFTPQC	SLIRSLTPTN	-NLLSQQQQQ	----------	------QQQQ	499
pig-tailed macaque (XP_011764131)	PQGH-LMSAL	PASNPGPSPP	YRPEKLSSPG	LPQQSFTPQC	SLIRSLTPTN	-NLLSQQQQQ	----------	------QQQQ	499
sooty mangabey (XP_011936741)	PQGH-LMSAL	PASNPGPSPP	YRPEKLSSPG	LPQQSFTPQC	SLIRSLTPTS	-NLLSQQQQQ	----------	------QQQQ	499
green monkey (XP_007991156)	PQGH-LMSAL	PASNPGPSPP	YRPEKLSSPG	LPQQSFTPQC	SLIRSLTPTS	-NLLSQQRQQ	----------	------QQQQ	499
white-tufted-ear marmoset (XP_008988264)	PQGH-LMSAL	PTSNPGPSPP	YRPEKLSSPG	LPQQSFTPQC	SLIRSLTPTS	-NLLSQQQQQ	QQ--------	------QQQQ	503
small-eared galago (XP_003800989)	PQGH-LMSAL	PASNPGPSPP	YCPEKLSSPG	LPQQSFTPQC	SLVRSVTPTS	-TLLGQQQ--	----------	------QQQQ	475
Sunda flying lemur (XP_008574565)	PPGH-LMSAL	PTSNPGPSPP	YRPEKLSSPG	LPQQSFTPQC	SLIRSLTPSN	-NLLSQ----	----------	------QQQQ	468
Chinese tree shrew (XP_006171265)	PQGHHLMSSL	PSSNPGPSPP	YRPEKLSSPG	LPQQSFTPQY	SLIRSLTPTS	-SLLSQQQPQ	PQPQ------	----------	470
Cape golden mole (XP_006874840)	PQGH-LMSAL	PTSNPGPSPP	YRPEKLSSPG	LPQQSFTPQC	SLLRSLTPTS	-TPLS-----	----------	----------	465
thirteen-lined ground squirrel (XP_005338579)	PQGH-LMSAL	PTSNPGPSPP	YRPEKLSSPG	LPQQSFTPQY	SLIRSLPPTS	TQLSQQQQQQ	Q---------	------QQQQ	476
rabbit (XP_008273259)	PQGH-LMSAL	PTNTPGPSPP	YRPEKLASPG	LPQQSFTPQC	SRIRSLTPTS	----------	----------	---------H	463
American pika (XP_004598606)	PQGH-LMSAL	PTSTLGPSPP	YRSEKLASPG	LPQQSFTLQC	SRIQNLMPTS	----------	----------	----------	462
house mouse (NP_001074823 XP_903797)	PQGH-LISAL	PTSTPGPSPP	YRPENLSSPG	LPQQSFTPQY	SLIRSLTPTS	-NLLSQQQQQ	QQ----QQQQ	--QQQQQQQQ	489
prairie deer mouse (XP_006973990)	PQGH-LISAL	PTSTPGPSPP	YRPENLSSPG	LPQQSFTPQY	SLIRSLTPTS	-NLLSQQQQQ	QQ----Q---	----QQQQQQ	485
Chinese hamster (XP_007629791)	PQGH-LISAL	PTSTPGPSPP	YRPENLSSPG	LPQQSFTPQY	SLIRSLTPTS	-NLLSQQQQQ	QQ----QHHH	HHQQQQQQQQ	492
prairie vole (XP_005369242)	PQGH-LLSAV	PTSTPGPSPP	YRPENLSSPG	LPQQSFTPQY	SLIKSLTPTS	-SLLSQQQQQ	QQ----Q---	--QQQHQLQH	487
Damara mole-rat (XP_010637336)	PQGH-LMSAL	PTSNPGPSPP	YRPEKLSSPG	LPQQSFTPQH	SLIRTLTPTS	-NLLSQQQQQ	QQHQQHQQHQ	QHQHQQQQQQ	486
lesser Egyptian jerboa (XP_004668990)	PQGH-LMSAL	PTSTPGPSPP	YRPEKLSSPG	LPQQSFTPQY	SLIRSLTPTS	-NLLSQQQQQ	QQQQQQQQQQ	QQQQQQQQQQ	487
big brown bat (XP_008156940)	PQGH-LMSAL	PTSNPGPSPP	YRPEKLSSPG	LPQQSFTPQC	SLIRSLTPSS	-NPLS-----	----------	----------	462
horse (NP_001166929 XP_001505137)	PQGH-LMSAL	PTSNPGPSPP	YRPEKLSSPG	LPQQSFTPQC	SLLRSLTPAS	-NPLSQQQ--	----------	------QQQQ	467
Bactrian camel (XP_010954813)	PQGH-LMSAL	PTSTPGPATP	YRPEKLSSPG	LPQQSFSPQC	SLIRSLTPTS	-NPLSQ----	----------	------PPPL	466
dog (XP_005641990)	PPGH-LLSAL	PTSNPGPSPP	YRPEKLSSPG	LPQQSFTPQC	SLIRSLTPSS	SNPLAQ----	----------	--PPPPPPPP	495
alpaca (XP_006216256)	PQGH-LMSAL	PTSNPGPTTP	YRPEKLSSPG	VPQQSFTPQC	SLIRSLTPTS	-NPLSQ----	----------	------PPPL	489
pig (XP_003135512)	PQGH-LMSAL	PTSTPGPATP	YRPEKLASPG	LPQQSFTPQC	PLVRSLTPTT	-HPLS-----	----------	----------	458
southern white rhinoceros (XP_004443252)	PQGH-LMPAL	PTSNPGPSPP	YRPEKLSSPG	LPQQSFTPQC	SLIRSLTPTS	-NPLSQQP--	----------	------QQQQ	465
Pacific walrus (XP_004407178)	PPGH-LLSAL	PTSTPGPSPP	YRPEKLSSPG	LPQQSFTPQC	SLIRSLTPSG	-NPLTQ----	----------	--PPPPPPPA	475
bottlenosed dolphin (XP_004325294)	PQGH-LISTL	PTSNPGPSTP	YRPEKLSSPG	LPQQSFTPQC	PLIRSLTPTS	-NPLGPQQ--	----------	-----PQLQL	491
Yangtze River dolphin (XP_007452175)	PQGH-LISTL	PTSNPGSSTP	YRPEKLSSPG	LPQQSFTPQC	PLIRSLTPTS	-NPLGPQQ--	----------	-----PQLQL	469
Florida manatee (XP_004390770)	PQGH-LMSAL	PTSTPGPSPP	YRPEKLSSPG	LPQQSFTPQC	SLLRSLTPTS	-TPLS-----	----------	----------	465
killer whale (XP_004284138)	PQGH-LISTL	PTSNLGPSTP	YRPEKLSSPG	LPQQSFTPQC	PLIRSLTPTS	-NPLGPQQ--	----------	-----PQLQL	493
sperm whale (XP_007129626)	PQGH-LISTL	PASNPGPSTP	YRPEKLSSPG	LPQQSFTPQR	PPIRSLAPTS	-SPLGPQQ--	----------	-----PQLQL	462
sheep (XP_011962574)	PQGH-LMSAL	PTSNTGPLTP	YHPETLSSPG	LPQQSFTAQC	PLIQGLT-TS	THPLC-----	----------	----------	463
goat (XP_005700529)	PQGH-LMSAL	PTSNTGPLTP	YRPETLSSPG	LPQQSFTAQC	PLIQGLT-TS	THPLC-----	----------	----------	461
chiru (XP_005962515)	PQGH-LMSAL	PTSNTGPLTP	YRPETLSSPG	LPQQSFTAQC	PLIQGLT-TS	THPLC-----	----------	----------	461
cattle (XP_010798654)	PQGH-LMSAL	PTSNTGPLTP	YRPETLSSPS	LPQQSFTAQC	PLIQGLT-TS	AHPLC-----	----------	----------	463
water buffalo (XP_006059754)	PQGH-LMSAL	PTSNTGPLTP	YRPETLSSPS	LPQQSFTAQC	PLIQGLT-TS	THPLC-----	----------	----------	463
nine-banded armadillo (XP_004448017)	PQGH-LMSAL	PTSNPGPSPP	YRPEQLSSPG	LPQQSFTPQC	SVTRGLAPTS	-NPLS-----	----------	------QQQQ	463

Supplemental Figure 1

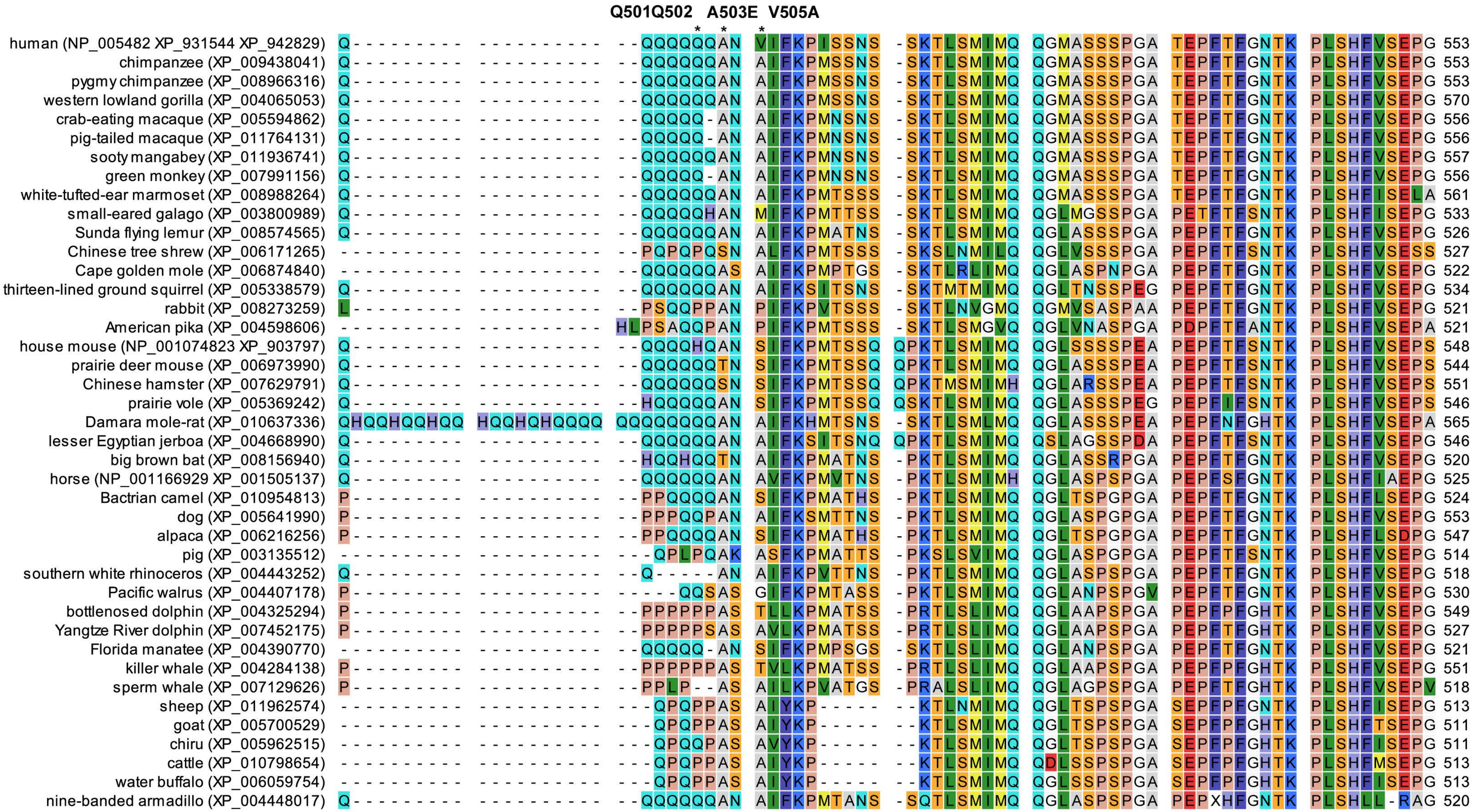

Supplemental Figure 1

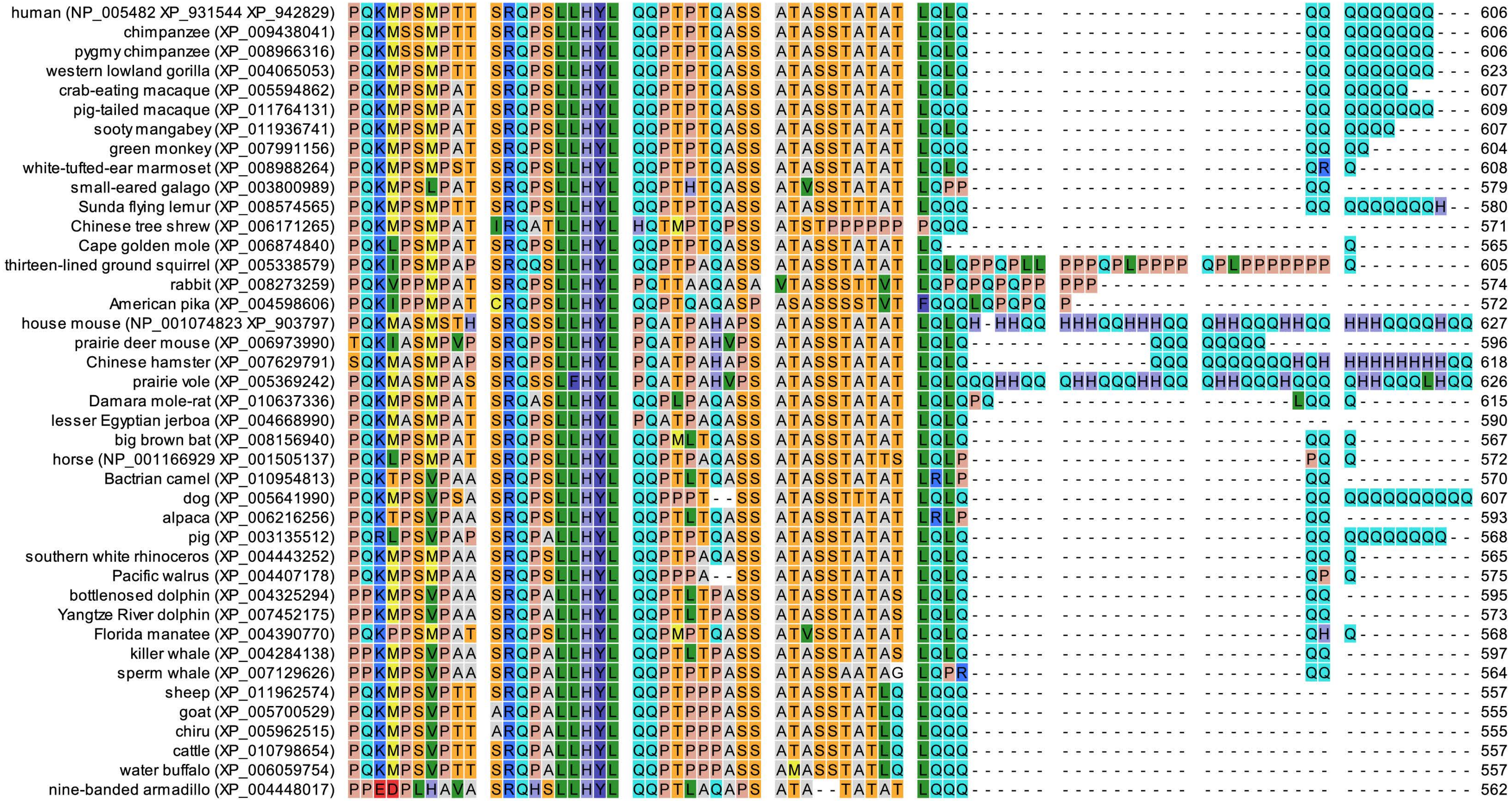

Supplemental Figure 1

human (NP_005482 XP_931544 XP_942829)	---------- -PDHSSFLLQ QMMQQPQRFQ RSVAS-DSMP ALPRQGCCHL F--------- ---AWTSAAS SVKP------	656
chimpanzee (XP_009438041)	---------- -PDHSSFLLQ QMMQQPQRFQ RSVAS-DSMP ALPRQGCCHL F--------- ---AWTSAAS SVKP------	656
pygmy chimpanzee (XP_008966316)	---------- -PDHSSFLLQ QMMQQPQRFQ RSVAS-DSMP ALPRQGCCHL F--------- ---AWTSAAS SVKP------	656
western lowland gorilla (XP_004065053)	---------- -PDHSSFLLQ QMMQQPQRFQ RSVAS-DSMP ALPRQGCCHL F--------- ---AWTSAAS SVKP------	673
crab-eating macaque (XP_005594862)	---------- -PDHSSFLLQ QMMQQPQRFQ RSVAS-DSMP ALPRQGCCHL F--------- ---AWTSAAS SVKP------	657
pig-tailed macaque (XP_011764131)	---------- -PDHSSFLLQ QMMQQPQRFQ RSVAS-DSMP ALPRQGCCHL F--------- ---AWTSAAS SVKP------	659
sooty mangabey (XP_011936741)	---------- -PDHSSFLLQ QMMQQPQRFQ RSVAS-DSTP ALPRQGCCHL F--------- ---AWTSAAS SVKP------	657
green monkey (XP_007991156)	---------- -PDHSSFLLQ QMMQQPQRFQ RSVAS-DSMP ALPRQGCCHL F--------- ---AWTSAAS SVKP------	654
white-tufted-ear marmoset (XP_008988264)	---------- -PDHSSFLLQ QMMQQPQRFQ RSVAS-DPMP ALPRQGCCHL F--------- ---AWTSAAS SVKP------	658
small-eared galago (XP_003800989)	---------- -ADHSSFLLQ QMMQPP-RFQ RPGAS-DAMS TLPRQGCCHL F--------- ---AWTSAAS SV--------	626
Sunda flying lemur (XP_008574565)	---------- -PDHSSFLLQ QMMQQPQRFQ RSVAS-DSMP ALPRQGYCHL F--------- ---ARTSTAS SVEH------	630
Chinese tree shrew (XP_006171265)	---------- QTDHSSFLLQ QMMQQPQRFQ RSVAS-DSTP ALPRQGSSYL F--------- ---AWTSAAS SV--------	620
Cape golden mole (XP_006874840)	---------- -PDHSSFLLQ QMIQQPQRLP RS------LP SEPRQACCHL F--------- ---AWTSAAS SV--------	608
thirteen-lined ground squirrel (XP_005338579)	---------- -PDHSAFLLQ QMMQQSQRFQ RSVAS-DSMP ALPRQCCCYL F--------- ---AWTSAA- ----------	650
rabbit (XP_008273259)	---------- -PDSSPFLLR QVVQQPQRFR RSVAA-DSVP ALPTQGCSQQ F--------- ---TWTSAAS SV--------	622
American pika (XP_004598606)	----KLQSRP QPDQSSFLLQ QVMQQTRRFQ RPVAS-DPAP ALPRQGCCHL F--------- ---TWNSAAS SV--------	627
house mouse (NP_001074823 XP_903797)	QQHQQ-QQQQ QPDQSSFLLQ QIMQQPQRFQ RMMAS-DSMP ALPGQGCCH- ---------- -RCAWTSTA- ----------	682
prairie deer mouse (XP_006973990)	---------- -SDQSTFLLQ QIMQQPQRFQ RMVTS-DSMS ALPGQGCCH- ---------- -RCAWTTAS- ----------	641
Chinese hamster (XP_007629791)	QQ-----QQQ QADQSSFLLQ HIMQQPQRFQ RMVAS-DSMP ALPGQGCCH- ---------- -RCAWTTAA- ----------	669
prairie vole (XP_005369242)	QQHHHHQQQQ QPDQSSFLLQ QIMQQPQRFQ RMVAS-DSMP ALPGQGCCH- ---------- -RCAWTTAA- ----------	682
Damara mole-rat (XP_010637336)	---------- -PEH-SFLLQ QMMQQPQRFQ RSVAS-ESVP TLPRQ----- ---------- ---------- ----------	647
lesser Egyptian jerboa (XP_004668990)	------QQLQ QSDQSSFLLQ QIMQQPQRFQ RLVAS-ESVP ALPGQGCCH- ---------- -PHAWTTDV- ----------	640
big brown bat (XP_008156940)	---------- -PDCSSFLLQ QMMQQPQRFQ RSAAS-ESMP PLLRKVRQPY IWLGSLVFGS HVLSWTSAAV SVE-------	628
horse (NP_001166929 XP_001505137)	---------- -PDHSAFLLQ QMMQQPQRFP RSGAS-DSMP SLPRQACCHL F--------- ---SWTSAAG S---------	619
Bactrian camel (XP_010954813)	---------- -PDQSSFLLQ QMMQQPQRFP RSVAS-EAMP SLPRQACCHL F--------- ---SWTSAAG S---------	617
dog (XP_005641990)	QQHQQHQQHQ QPDHSSFLLQ QMMQQPQRFQ RSVAP-DSMP SLPRQ----- ---------- ---------- ----------	651
alpaca (XP_006216256)	---------- -PDQSSFLLQ QMMQQPQRFP RSVAS-EAMP SLPRQACCHL F--------- ---SWTSAAG S---------	640
pig (XP_003135512)	---------- -PDHSSFLLQ QVMQQPQRLQ RSVAS-DAVP SLPRQACCHL F--------- ---SWTSAAG S---------	615
southern white rhinoceros (XP_004443252)	---------- -PDHSSFLLQ QMMQQPQRFP RSVAS-DSMP SLPRQSPLFV VLIPAYVSYV LPRSW-SFDG STQPAVLTRG	632
Pacific walrus (XP_004407178)	---------- -PDHSSFLLQ QVMQQPQRFQ RSAAP-DSMA SLPRQMPLII ALAPVRTGYL LP-------- ----------	625
bottlenosed dolphin (XP_004325294)	---------- -PDAASFLLQ HVTQQPQRVQ RSVAS-DSMP SLPRQACCQL F--------- ---SWTSAAG ----------	641
Yangtze River dolphin (XP_007452175)	---------- -PDAASFLLQ HVTQQPQRVQ RSVAS-DSMP SLPRQACCQL F--------- ---SWTSAAG ----------	619
Florida manatee (XP_004390770)	---------- -PDHSSILLQ QMIQQPQRLP RS------LP SEPRQACCHL F--------- ---AWTSAAS SV--------	611
killer whale (XP_004284138)	---------- -PDAASFLLQ HVTQQPQRVQ RSVAS-DSMP SLPRQACCQL F--------- ---SWTSAAG ----------	643
sperm whale (XP_007129626)	---------- -PDAASFLLQ HVTQQPRRVQ RSVAS-ESVP SLPRRACCQL F--------- ---SWTSAAG ----------	610
sheep (XP_011962574)	---------- -PDLSSFLLQ HVTQQPQRFQ RSLASGDSMP SLPRQACCQL F--------- ---SWTSVAG SMEHQL----	610
goat (XP_005700529)	---------- -PDLSSFLLQ HVTQQPQRFQ RSLASGDSMP SLPRQACCQL F--------- ---SWTSVAG SMEHQL----	608
chiru (XP_005962515)	---------- -PDLSSFLLQ HVTQQPQRFQ RSLASGDSMP SLPRQACCQL F--------- ---SWTSVAG SMEHQL----	608
cattle (XP_010798654)	---------- -PDISSFLLQ HVTQQPQRFQ RSLALGDSMP SLPRQACCQL F--------- ---SWTSVAG SMEHQL----	610
water buffalo (XP_006059754)	---------- QPDLSSFLLQ HVTQQPQRFQ RSLALGDSMP SLPRQACCQL F--------- ---SWTSMAG SMEHQL----	611
nine-banded armadillo (XP_004448017)	---------- -PDESSFLLQ QMMQQPQRFQ RATAL-DSMP SLPRQAGCHL F--------- ---AWTSAAS SV--------	610

Supplemental Figure 1

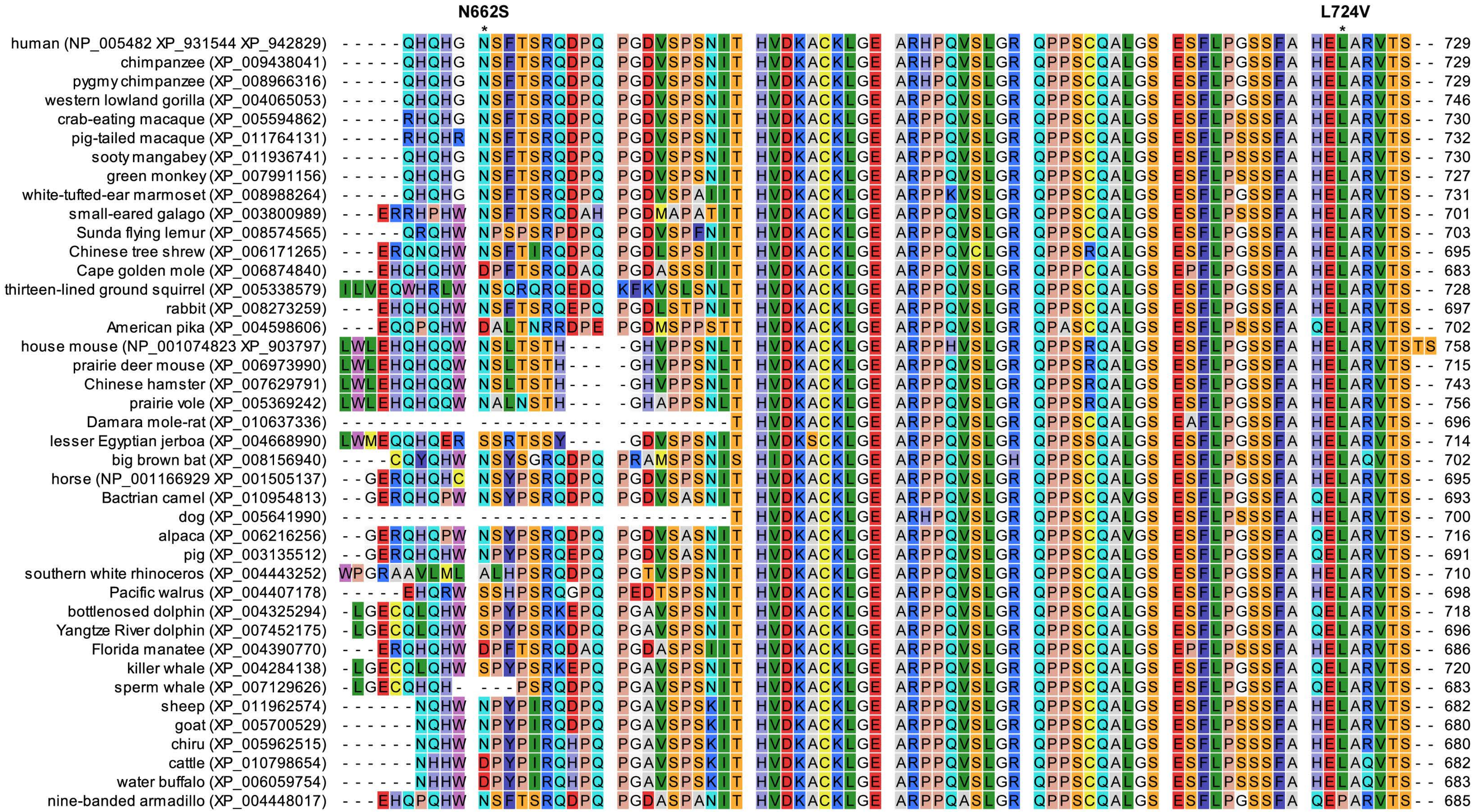

Supplemental Figure 1

S730S

Species	*					
human (NP_005482 XP_931544 XP_942829)	SYSTSEAAPW	GSWDPKAWRQ	-VPAPLLPSC	DATARGTEIR	SYGNDP	774
chimpanzee (XP_009438041)	SYSTSEAAPW	GSWDPKAWRQ	-VPAPLLPSC	DATARGTEIR	SYGNDP	774
pygmy chimpanzee (XP_008966316)	SYSTSEAAPW	GSWDPKAWRQ	-VPAPLLPSC	DATARGTEIR	SYGNDP	774
western lowland gorilla (XP_004065053)	SYSTSEAAPW	GSWDPKAWRQ	-VPAPLLPSC	DTTARGTEIR	SYGNDP	791
crab-eating macaque (XP_005594862)	SYSTSEAAPW	GSWDPKAWRQ	-VPAPLLPSC	DAAARGTEIR	SYGNDP	775
pig-tailed macaque (XP_011764131)	SYSTSEAAPW	GSWDPKAWRQ	-VPAPLLPSC	DAAARGTEIR	SYGNDP	777
sooty mangabey (XP_011936741)	SYSTSEAAPW	GSWDPKAWRQ	-VPAPLLPSC	DAAARGTEIR	SYGNDP	775
green monkey (XP_007991156)	SYSTSEAAPW	GSWDPKAWRQ	-VPAPLLPSC	DAAARGTEIR	SYGNDP	772
white-tufted-ear marmoset (XP_008988264)	SYSTSEAAPW	GSWDPKAWRQ	-VPAPLLPSC	DAAARETEIR	SYGNDP	776
small-eared galago (XP_003800989)	SYSTSEAAPW	GSWDPKAWRQ	-VPAPLLPSC	DAAAREAEIR	SYGNDP	746
Sunda flying lemur (XP_008574565)	SYSTSEAAPW	GGWDPKAWRQ	-VPAPLLPSC	DAAAREAEIR	SYGNDP	748
Chinese tree shrew (XP_006171265)	SYSTSEAAPW	GGWDPKAWRQ	-VPAPLLPSC	DAAAREAEIR	SYGNDP	740
Cape golden mole (XP_006874840)	SHSTSEAAPW	GGWDPKAWRQ	-VPAPLLPSC	EATARETETR	SYGNDP	728
thirteen-lined ground squirrel (XP_005338579)	SYSTSEAAPW	GGWDPKDWRQ	-VPAPLLPSC	DAAARETEIR	SYGNNP	773
rabbit (XP_008273259)	-YSTSEAAPW	GSWDPKPWRQ	-VPAPLLPSC	EAAARETEIR	SYGNDP	741
American pika (XP_004598606)	-YSTSEAAPW	GGWDPKAWRQ	-VPDPLLPSC	AAAAREAEIR	SYGNDP	746
house mouse (NP_001074823 XP_903797)	SYNTSEAAPW	GGWDPKAWRQ	-VPAPLLPSC	DAAAREAEIR	SYGNDP	803
prairie deer mouse (XP_006973990)	SYSTSEAAPW	GGWDPKAWRQ	-VPAPLLPSC	DAAAKEAEIR	SYGNDP	760
Chinese hamster (XP_007629791)	SYSTSEAAPW	GGWDPKAWRQ	-VPAPLLPSC	DAAAREAEIR	SYGNDP	788
prairie vole (XP_005369242)	SYGTSEAAPW	GSWDPKAWRQ	-VPAPLLPSC	DAAAREAEIR	SYGNDP	801
Damara mole-rat (XP_010637336)	SYSTSEAAPW	GGWDPKAWRQ	-VPASLLPSC	DATARETEIR	SYGNDP	741
lesser Egyptian jerboa (XP_004668990)	SYNTSEAAPW	GGWDPKAWRQ	-VPAPLLPSC	DAAAREAEIR	SYGNDP	759
big brown bat (XP_008156940)	SYSTSEAAPW	GGWDPKAWRQ	-VPAPLLPSC	DAVARETEIR	SYGNDP	747
horse (NP_001166929 XP_001505137)	SYSTSEAAPW	GGWDPKAWRQ	-VPAPLLPSC	DAVARETEIR	SYGNDP	740
Bactrian camel (XP_010954813)	SYGTSEAAPW	GGWDPKAWRQ	-VPAPLLPSC	DAVAREAEIR	SYGNDP	738
dog (XP_005641990)	SHSTSEAAPW	GGWDPKAWRQ	-VPAPLLPSC	DAVAREAEIR	SYGNDP	745
alpaca (XP_006216256)	SYGTSEAAPW	GGWDPKAWRQ	-VPAPLLPSC	DAVAREAEIR	SYGNDP	761
pig (XP_003135512)	SYSTSEAAPW	GGWDPKAWRQ	QVPAPLLPRC	DAVARETEIR	SYGNDP	737
southern white rhinoceros (XP_004443252)	SYSTSEAAPW	GGWDPKAWRQ	-VPAPLLPSC	DAVAREAEIR	SYGNDP	755
Pacific walrus (XP_004407178)	SYSTSEAAPW	GGWDPKAWRQ	-VPAPPLPSC	DAVAREAEIR	SYGNDP	743
bottlenosed dolphin (XP_004325294)	SYSTSEAAPW	GGWDPKAWRQ	-VPAPLLPSC	DAVAREAEIR	SYGNDP	763
Yangtze River dolphin (XP_007452175)	SYSTSEAAPW	GGWDPKAWRQ	-VPAPLLPSC	DAVGREAEIR	SYGNDP	741
Florida manatee (XP_004390770)	SHSTSEAAPW	GGWDPKAWRQ	-VPAPLLPSC	DAAARETESR	SYGNDP	731
killer whale (XP_004284138)	SYSTSEAAPW	GGWDPKAWRQ	-VPAPLLPSC	DAVAREAEIR	SYGNDP	765
sperm whale (XP_007129626)	SYSTSEAAPW	GGWDPKAWRQ	-VPAPLLPSC	DAVAREAEIR	SYGNDP	728
sheep (XP_011962574)	SYSTSEAAPW	GGWDPKAWRQ	-VPAPLLPSC	DAVAREAEIR	SYGNDP	727
goat (XP_005700529)	SYSTSEAAPW	GGWDPKAWRQ	-VPAPLLPSC	DAVAREAEIR	SYGNDP	725
chiru (XP_005962515)	SYSTSEAAPW	GGWDPKAWRQ	-VPAPLLPSC	DAVAREAEIR	SYGNDP	725
cattle (XP_010798654)	SYSTSEAAPW	GGWDPKAWRQ	-VPAPLLPSC	DTVAREAEIR	SYGNDP	727
water buffalo (XP_006059754)	SYSTSEAAPW	GGWDPKAWRQ	-VPAPLLPSC	DTVAREAEIR	SYGNDP	728
nine-banded armadillo (XP_004448017)	SHSASEAAPW	GGWDPKAWRQ	-VPAPPLPSC	DAAAREAELR	SYGNDP	730
