# Supplementary figures and images for: Human MAMLD1 Gene Variations Seem Not Sufficient to Explain a 46,XY DSD Phenotype
Source: PLoS One. 2015 Nov 16;10(11):e0142831. doi: 10.1371/journal.pone.0142831 (PMC4646284; doi:10.1371/journal.pone.0142831)

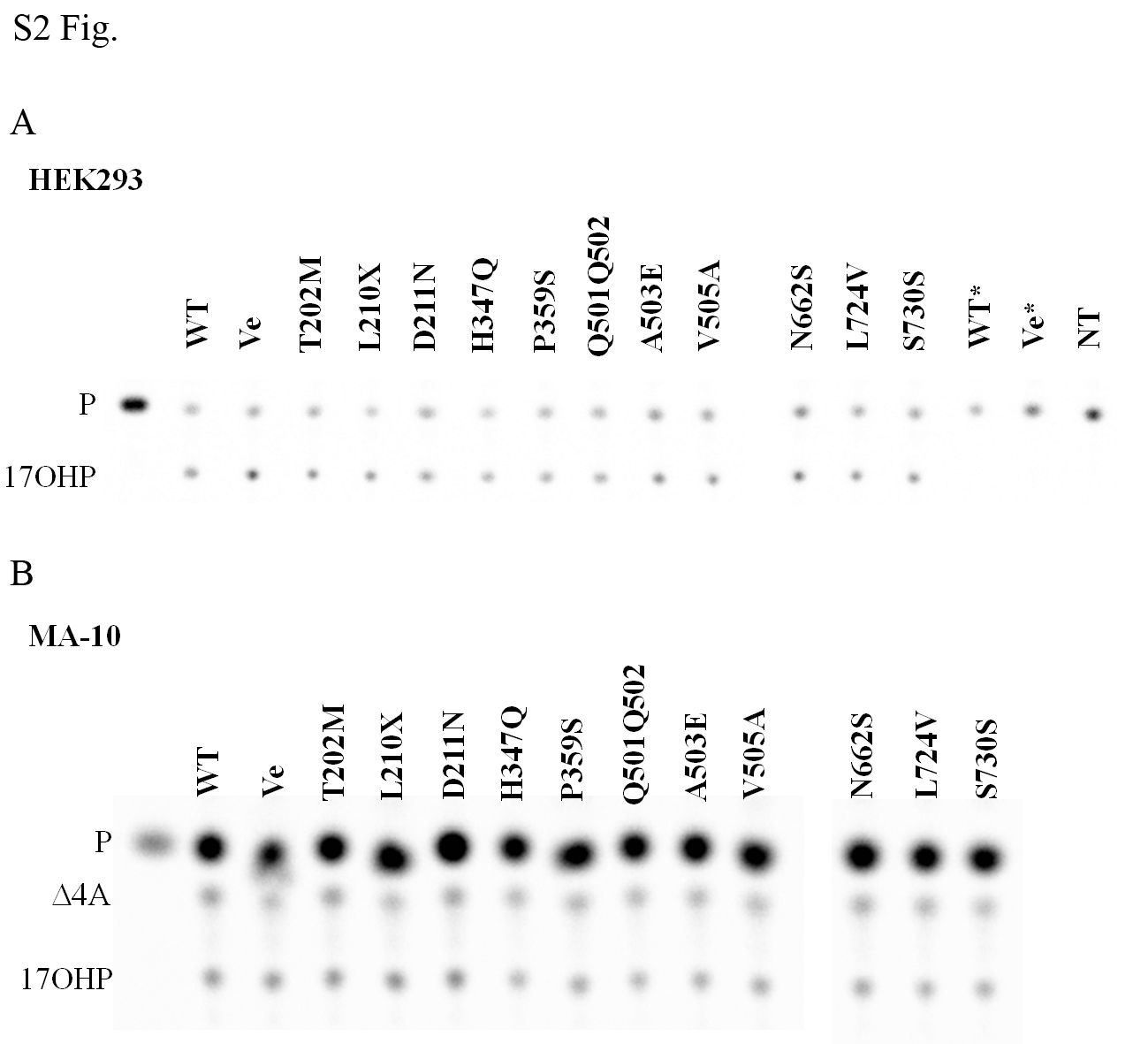

Supplement: S2 Fig — Cells were transiently transfected with MAMLD1 WT and mutant expression vectors. HEK293 cells were also co-transfected with the CYP17A1 expression vector as they do not express it endogenously. The effect of WT and mutant MAMLD1 on CYP17A1 enzyme activity was assessed by measuring the conversion of progesterone (P) to 17-hydroxyprogesterone (17OHP) in non-steroidogenic HEK293 cells, and conversion of P to 17OHP and then to androstenedione (Δ4A) in steroidogenic mouse Leydig MA-10 cells. Steroid production was labeled with [14C]progesterone for 60 min. Steroids were extracted and resolved by thin-layer chromatography, then quantified as % conversion. A representative steroid profile obtained from HEK293 (A) and MA-10 (B) cells is shown (n = 2). Similar to experiments performed in NCI-H295R cells (Fig 3B), no effect of MAMLD1 on CYP17-hydroxylase activity was detected. Ve: empty vector; WT: wild type; *: co-transfected with empty vector; NT: non-transfected. (TIF) [file pone.0142831.s002.tif]

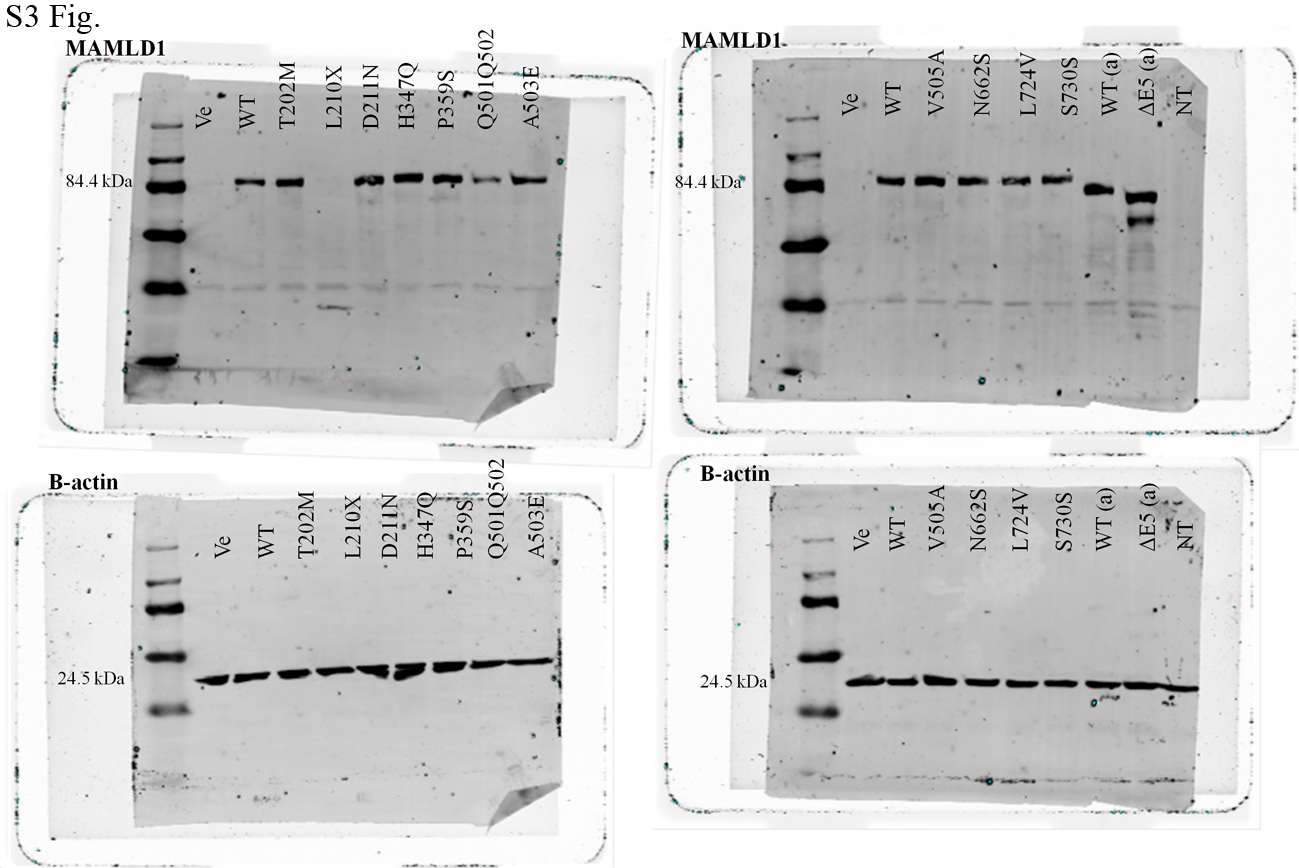

Supplement: S3 Fig — Picture of the original uncropped and unadjusted Western blots for myc-MAMLD1 (A) and actin (B) corresponding to Fig 4. Ve: empty vector; WT: wild type; NT: non-transfected. (TIF) [file pone.0142831.s003.tif]
