# Supplementary material for: Human MAMLD1 Gene Variations Seem Not Sufficient to Explain a 46,XY DSD Phenotype
Source: PLoS One. 2015 Nov 16;10(11):e0142831. doi: 10.1371/journal.pone.0142831 (PMC4646284; doi:10.1371/journal.pone.0142831)
Supplement: S1 Table — (DOCX) [file pone.0142831.s005.docx]

**Table S1.** Primers used in this study ^a^.

| **Gene amplification and sequencing** | | | |
| --- | --- | --- | --- |
| *Primer name* | *Sequence (5’🡪 3’)* | *Location* | *Product size* |
| MAMLD1-1s^b^ | TCACGGGGTCAGTGGTCTC | 5’UTR | 259 bp |
| MAMLD1-1a^b^ | CATTAAATACCCCCCTCCC | Intron 1 |  |
| MAMLD1-2s^b^ | GTGTGTGCAAGTGGCTCTC | Intron 1 | 182 bp |
| MAMLD1-2a^b^ | CACTCGGGGCCTCAGTTTC | Intron 2 |  |
| MAMLD1-3As^c^ | CTCTCTTCTCCTCTTCTCTTCTCT | Intron 2 | 416 bp |
| MAMLD1-3Aa^c^ | GGTGAGCTCCTCTAGCAGCTCTTG | Exon 3 |  |
| MAMLD1-3Bs^c^ | GCCGGCTGTAGACCAGGAGC | Exon 3 | 407 bp |
| MAMLD1-3Ba^c^ | GGGCGTGATGCCACTGAGGC | Exon 3 |  |
| MAMLD1-3Cs^c^ | GTCGCTCTGCCCCCCTTACC | Exon 3 | 412 bp |
| MAMLD1-3Ca^c^ | GAATGGAGCTCTGAGGGCCG | Exon 3 |  |
| MAMLD1-3Ds^c^ | GCTCTCACTCAACAGCCGCAG | Exon 3 | 368 bp |
| MAMLD1-3Da^c^ | GGCTCCGTGGCTCCTGGGCTG | Exon 3 |  |
| MAMLD1-3Es^c^ | CATGATCATGCAGCAGGGGATGGCAAGC | Exon 3 | 401 bp |
| MAMLD1-3Ea^c^ | CCAAAACAAAAGAACACAGCCAGATATG | Intron 3 |  |
| MAMLD1-4s^c^ | TAGGACACGGCAGGCCACCTG | Intron 3 | 282 bp |
| MAMLD1-4a^c^ | AGCCAGCTGATCCAGCTCTGG | Intron 4 |  |
| MAMLD1-5s^c^ | TTTGTGGCCAAGCAGCTGATG | Intron 4 | 342 bp |
| MAMLD1-5a^c^ | GACTGTGCCCCGTTGACAGAT | Intron 5 |  |
| MAMLD1-6s^c^ | CTAGGTCCCCACGCAGCGATC | Intron 5 | 210 bp |
| MAMLD1-6a^c^ | ACCCCTTGCCCACCCCTTTGG | Intron 6 |  |
| **Vectors sequencing** | | | |
| *Primer name* | *Sequence (5’🡪 3’)* | *Location* | *Product size* |
| MAMLD1-F1 (258) | CTTGCCTTGAAGATGTCAC | c.258, exon 4 | - |
| MAMLD1-R1 (762) | CATGTCACTTCAGATCCCA | c.762, exon 4 | - |
| MAMLD1-F2 (837) | ACAGTCCAAGAGCCAGGT | c.837, exon 4 | - |
| MAMLD1-F3 (1663) | CAGAAGATGCCCTCCATG | c.1663, exon 4 | - |
| **Gene expression** | | | |
| *Primer name* | *Sequence (5’🡪 3’)* | *Location* | *Product size* |
| MAMLD1 (1-582)_F | 5’ g gcc cGA ATT Cgg ATG GAT GAC TGG AAA AGT | c.1, exon 2 | 582 bp |
| MAMLD1 (1-582)_R2 | 5‘ gAG ATC TAG CTC ATT TGG AGA AGG | c.582, exon 4 | 582 bp |
| GAPDH F | GTA TCG TGG AAG GAC TCA T | Exon 7 | 500 bp |
| GAPDH R | TAC TCC TTG GAG GCC ATG T | Exon 9 | 500 bp |
| **Site-directed mutagenesis** | | |  |
| *Primer name* | *Sequence (5’🡪 3’)* | *Change and location* |  |
| MAMLD1-T202M_F: | GAAGATACTGGGGATGAAGCCAGAAGAGC | c.605C>T, exon 4 |  |
| MAMLD1-T202M_R: | GCTCTTCTGGCTTCATCCCCAGTATCTTC | c.605C>T, exon 4 |  |
| MAMLD1-L210X_F: | CAGAAGAGCCACTGG TTTAGATCATCCCCA | c.626delT, exon 4 |  |
| MAMLD1-L210X_R: | TGGGGTTGATCTTTT CCAGTGGCTCTTCTG | c.626delT, exon 4 |  |
| MAMLD1-D211N_F: | AGCCACTGGTTTTAAATCATCCCCAGGCA | c.631G>A, exon 4 |  |
| MAMLD1-D211N_R: | TGCCTGGGGATGATTTAAAACCAGTGGCT | c.631G>A, exon 4 |  |
| MAMLD1-H347Q_F: | GTGCCATCACCACAACCACCACCGCTGCC | c.1041C>A, exon 4 |  |
| MAMLD1-H347Q_R: | GGCAGCGGTGGTGGTTGTGGTGATGGCAC | c.1041C>A, exon 4 |  |
| MAMLD1-P359S_F: | CACCACCACCACCCTCATTCAGCCCCCAG | c.1075C>T, exon 4 |  |
| MAMLD1-P359S_R: | CTGGGGGCTGAATGAGGGTGGTGGTGGTG | c.1075C>T, exon 4 |  |
| MAMLD1-A503E_F: | GCAGCAGCAGCAAGAAAATGTGATCTTTA | c.1508C>A, exon 4 |  |
| MAMLD1-A503E_R: | TAAAGATCACATTTTCTTGCTGCTGCTGC | c.1508C>A, exon 4 |  |
| MAMLD1-V505A_F: | GCAGCAAGCAAATGCGATCTTTAAGCCCA | c.1514T>C, exon 4 |  |
| MAMLD1-V505A_R: | TGGGCTTAAAGATCGCATTTGCTTGCTGC | c.1514T>C, exon 4 |  |
| MAMLD1-N662S_F: | GCATCAACACGGGAGCTCTTTCACTAGCA | c.1985A>G, exon 5 |  |
| MAMLD1-N662S_R: | TGCTAGTGAAAGAGCTCCCGTGTTGATGC | c.1985A>G, exon 5 |  |
| MAMLD1-L724V_F: | CCTTTGCTCATGAGGTGGCCCGAGTCACC | c.2170C>G, exon 6 |  |
| MAMLD1-L724V_F: | GGTGACTCGGGCCACCTCATGAGCAAAGG | c.2170C>G, exon 6 |  |
| MAMLD1-S730S_F: | CGAGTCACCTCCTCATACAGCACCTCAGA | c.2190G>A, exon 6 |  |
| MAMLD1-S730S_R: | TCTGAGGTGCTGTATGAGGAGGTGACTCG | c.2190G>A, exon 6 |  |

^a^ GeneBank entries for the design of the primers: MAMLD1: isoform 2 NM_005491.3, CCDS14693.2; GAPDH: NM_002046.5.

Underlined: mutation.

^b^ Primers design by us.

^c^ Primers from Kalfa *et al*. [[1](#_ENREF_1)]

**References**

Kalfa N, Liu B, Klein O, Audran F, Wang MH, et al. (2008) Mutations of CXorf6 are associated with a range of severities of hypospadias. European journal of endocrinology / European Federation of Endocrine Societies 159: 453-458.
